# Paclitaxel-Coated versus Uncoated Balloon for Femoropopliteal In-Stent Restenosis: A Systematic Review and Meta-Analysis

**DOI:** 10.31083/j.rcm2309315

**Published:** 2022-09-14

**Authors:** Qiwei Li, Li Wang, Lu Zhu, Yong Wu, Limin Wu, Hanmin Liu

**Affiliations:** ^1^Department of Pediatric Pulmonology and Immunology, West China Second University Hospital, Sichuan University, 610041 Chengdu, Sichuan, China; ^2^Key Laboratory of Birth Defects and Related Diseases of Women and Children (Sichuan University), Ministry of Education, 610041 Chengdu, Sichuan, China; ^3^NHC Key Laboratory of Chronobiology (Sichuan University), 610041 Chengdu, Sichuan, China; ^4^The Joint Laboratory for Lung Development and Related Diseases of West China Second University Hospital, Sichuan University and School of Life Sciences of Fudan University, West China Institute of Women and Children’s Health, West China Second University Hospital, Sichuan University, 610041 Chengdu, Sichuan, China; ^5^Sichuan Birth Defects Clinical Research Center, West China Second University Hospital, Sichuan University, 610041 Chengdu, Sichuan, China; ^6^Department of Pediatrics, The Affiliated Hospital of Southwest Medical University, 646000 Luzhou, Sichuan, China; ^7^Sichuan Clinical Research Center for Birth Defects, 646000 Luzhou, Sichuan, China; ^8^Mianyang Central Hospital, School of Medicine, University of Electronic Science and Technology of China, 621000 Mianyang, Sichuan, China; ^9^Department of Orthopaedics, Orthopedic Research Institute, West China Hospital, Sichuan University, 610041 Chengdu, Sichuan, China

**Keywords:** lower extremity artery disease, femoropopliteal artery, in-stent restenosis, paclitaxel, drug-coated balloon

## Abstract

**Background:**

Several prospective controlled trials to date have assessed the safety and efficacy of paclitaxel-coated balloon angioplasty (PCBA) versus uncoated balloon angioplasty (UCBA) for femoropopliteal (FP) in-stent restenosis (ISR). Therefore, this meta-analysis of prospective controlled trials aimed to summarize the results of these trials and present reliable conclusions.

**Methods:**

We systematically searched the PubMed, Embase, Cochrane Library, Web of Science, ClinicalTrials.gov, and CNKI databases for prospective randomized controlled trials (published between January 1, 2008, and July 31, 2021; no language restrictions) comparing PCBA with UCBA in the management of FP ISR. The main endpoints were recurrent restenosis, primary patency, freedom from target lesion revascularization (TLR), clinical improvement, ankle-brachial index (ABI), and major adverse events (MAEs). We assessed the pooled data using a fixed effects model.

**Results:**

Of the 206 identified studies, seven were eligible and included in our analysis (N = 593 participants). Compared with UCBA, PCBA yielded a reduction in recurrent restenosis (odds ratio [OR], 0.22; 95% confidence interval [CI], 0.13–0.38), a better primary patency (OR, 3.59; 95% CI, 1.72–7.47), an improved likelihood of freedom from TLR (OR, 2.70; 95% CI, 1.36–5.35), greater clinical improvement (OR, 2.38; 95% CI, 1.50–3.79), and a similar mean difference in ABI (0.02; 95% CI, –0.11–0.14) and OR in MAEs (0.71; 95% CI, 0.24–2.14).

**Conclusions:**

PCBA as a treatment strategy can achieve better short-term outcomes of FP ISR management, including potent recurrent restenosis-lowering and symptom-improving capacity without increased MAEs. Therefore, it is a promising therapeutic strategy for patients with FP ISR.

**Systematic Review Registration:**

This work was registered in PROSPERO, the international prospective register of systematic reviews (number: CRD42021261574).

## 1. Introduction

Approximately 202 million people worldwide live with lower extremity artery disease (LEAD) [1], a common cause of which is chronic obstruction of the femoropopliteal artery (FPA) [2]. Modern bare metal nitinol stents (BMSs) have been widely used to manage FP lesions; however, their long-term patency and durability in the FP region are suboptimal [3], while in-stent restenosis (ISR) is the main challenge related to their durability [4]. The 12-month ISR rates after BMS implantation in the superficial femoral artery (SFA) and proximal popliteal artery are 18–37%.

Several methods have been applied to manage ISR, including percutaneous transluminal angioplasty (PTA), drug-coated balloon (DCB) implantation, and repeat stenting [5]. The advent of drug-eluting stent (DES) technology has reduced ISR rates but eventually leads to disappointment [6]. In recent years, paclitaxel (PTX) has been applied in DES and DCB because of its potent inhibitory effect on smooth muscle cell migration and proliferation at low concentrations. Furthermore, owing to the efficacious delivery of PTX from balloons, PTX-coated balloons (PCBs) have been developed as an alternative to DES and have been particularly successful in the treatment of peripheral artery disease (PAD) [7].

Last year, the DAEDALUS study compared PCB and DES for coronary ISR [8] and concluded that PCB for FP ISR is promising. To date, many trials have assessed the effectiveness and safety of PCBs for FP ISR, while horizontal comparisons of PCBs and uncoated balloon (UCB) are rare.

In the last five years, several new trials have been published; therefore, this meta-analysis aimed to assess the overall outcomes of prospective controlled trials comparing PCB with UCB for FP ISR.

## 2. Methods

### 2.1 Study Principle and Registration

This meta-analysis was reported in accordance with the Preferred Reporting Items for Systematic Reviews and Meta-analysis (PRISMA) Statement and Assessing the Methodological Quality of SysTemAtic Reviews (AMSTAR) guidelines [9]. The protocol of this analysis was registered in PROSPERO, the international prospective register of systematic reviews (number: CRD42021261574).

### 2.2 Search Strategy and Information Sources

We searched relevant studies published between January 1, 2008 (when the first study was published) and July 31, 2021 by searching the PubMed, Embase, Cochrane, Web of Science, ClinicalTrials.gov, and CNKI databases. No language restrictions were applied during this process. Unpublished but completed studies were also sought. The key terms used were “paclitaxel-coated balloon”, “in-stent restenosis”, and “femoropopliteal”. The complete search strategy used in PubMed was as follows: (paclitaxel[Title/Abstract]) AND (drug-coated balloon[Title/Abstract] OR drug-eluting balloon[Title/Abstract]) AND (uncoated balloon[Title/Abstract] OR angioplasty[Title/Abstract]) AND (in-stent restenosis[Title/Abstract]) AND (femoropopliteal[Title/Abstract] OR femoral[Title/Abstract] OR popliteal[Title/Abstract]).

### 2.3 Study Selection and Selection Criteria

We included eligible trials fulfilling the following criteria: (1) prospective randomized controlled design; (2) comparison of PCB and UCB in FP ISR; (3) single intervention for each group; (4) follow-up time >6 months; and (5) reporting on recurrent restenosis, primary patency, freedom from target lesion revascularization (TLR), clinical improvement, ankle-brachial index (ABI), and/or major adverse events (MAEs). Additionally, reviews and studies were excluded because: (1) data were unavailable for analysis; (2) the study objective was not related to ISR or PCB; (3) the intervention was combined rather than single; (4) the study design was retrospective; (5) the study was of animals; (6) no group comparisons were made; or (7) the study was a duplicate. All studies were collected into and included or excluded from EndNote (X8; Clarivate, London, United Kingdom). Two independent investigators reviewed the study titles and abstracts and those that fulfilled the inclusion criteria were retrieved for full-text assessment. Studies that qualified for full-text review were then reviewed by two independent investigators for inclusion in or exclusion from the analysis.

### 2.4 Data Extraction and Outcome Variables

In the data extraction process, one author extracted data from the included studies and another author verified their accuracy. The following data were extracted from the included studies: total number of participants, age, sex, trial duration, diabetes mellitus (DM), hypertension, coronary artery disease (CAD), smoking, obesity, chronic kidney disease (CKD), ABI, Rutherford class, ISR Tosaka classification, inclusion and exclusion criteria, intervention, follow-up duration, and outcomes.

Based on the European Society of Cardiology (ESC) guidelines for the diagnosis and treatment of PAD [3] and the specific conditions of the included studies, we chose recurrent restenosis, freedom from TLR, primary patency, clinical improvement, ABI, and MAEs as the outcomes of this meta-analysis. We set recurrent restenosis as the main outcome, MAEs as the safety outcome, and the other variables as additional outcomes.

Recurrent restenosis was defined as >50% stenosis on angiography or a duplex-derived peak systolic velocity ratio ≥2.5 within the treated arterial segment. Primary patency was defined as <50% stenosis on duplex ultrasonography (DUS) and computed tomographic angiography (CTA) in the absence of clinically driven TLR. Freedom from TLR meant that participants did not undergo revascularization during follow-up. Clinical improvement was defined as a post-treatment improvement of ≥1 Rutherford category. A post-treatment increase in ABI revealed hemodynamic improvement. MAEs included all-cause death, major amputation, and surgical intervention of the target limb.

### 2.5 Assessment of Risk of Bias and Heterogeneity

According to the Cochrane Handbook for Systematic Reviews of Intervention [10], we used the Cochrane risk of bias tool to assess the following: random sequence generation (selection bias), allocation concealment (selection bias), blinding of participants and personnel (performance bias), blinding of outcome assessment (detection bias), incomplete outcomes data (attrition bias), selective reporting (reporting bias), and other. We then created a risk of bias graph and risk of bias summary. Any disagreements were discussed by the entire group and eliminated.

Heterogeneity between the included studies was examined using the Cochrane Q and $I^{2}$ tests. In the Q test, values of *p *> 0.1 indicated no heterogeneity across studies. In the $I^{2}$ test, values over 50% were indicative of moderate to high heterogeneity based on the Cochrane Handbook. A sensitivity analysis was performed to determine the sources of heterogeneity and assess the stability of the results.

### 2.6 Statistical Analysis

We then assessed the effect of PTX-coated balloon angioplasty (PCBA) versus uncoated balloon angioplasty (UCBA) on six outcomes. Statistical analyses were performed on an intention-to-treat basis using Review Manager software (version 5.4; The Cochrane Collaboration, London, United Kingdom) and STATA (SE 15.0; StataCorp LLC, College Station, Texas, USA). We analyzed recurrent restenosis, primary patency, freedom from TLR, clinical improvement, and MAEs as dichotomous data, which were synthesized using the Mantel-Haenszel model, fixed-effects model, and odds ratios (ORs) with 95% confidence intervals (CIs). ABI was analyzed as continuous data and synthesized by the application of inverse variance, the random-effects model, and mean difference. ORs with 95% CIs and weighted mean differences (WMDs) were used as summary statistics and calculated by the models stated above.

Once the pooled ORs and WMDs were calculated, the sensitivity analysis was performed to determine the potential influence of each study on the overall meta-analysis estimates. Furthermore, according to the Grading of Recommendations Assessment, Development and Evaluation (GRADE) method, we used the GRADE profiler (version 3.6.1; GRADE Working Group, Hamilton, Ontario, Canada) to assess the quality of all studies included in this meta-analysis. We evaluated the possibility of publication bias by constructing a funnel plot and using the Begg and Egger’s tests, defining significant publication bias as values of *p *< 0.1.

## 3. Results

### 3.1 Study Selection

**Fig. 1.**
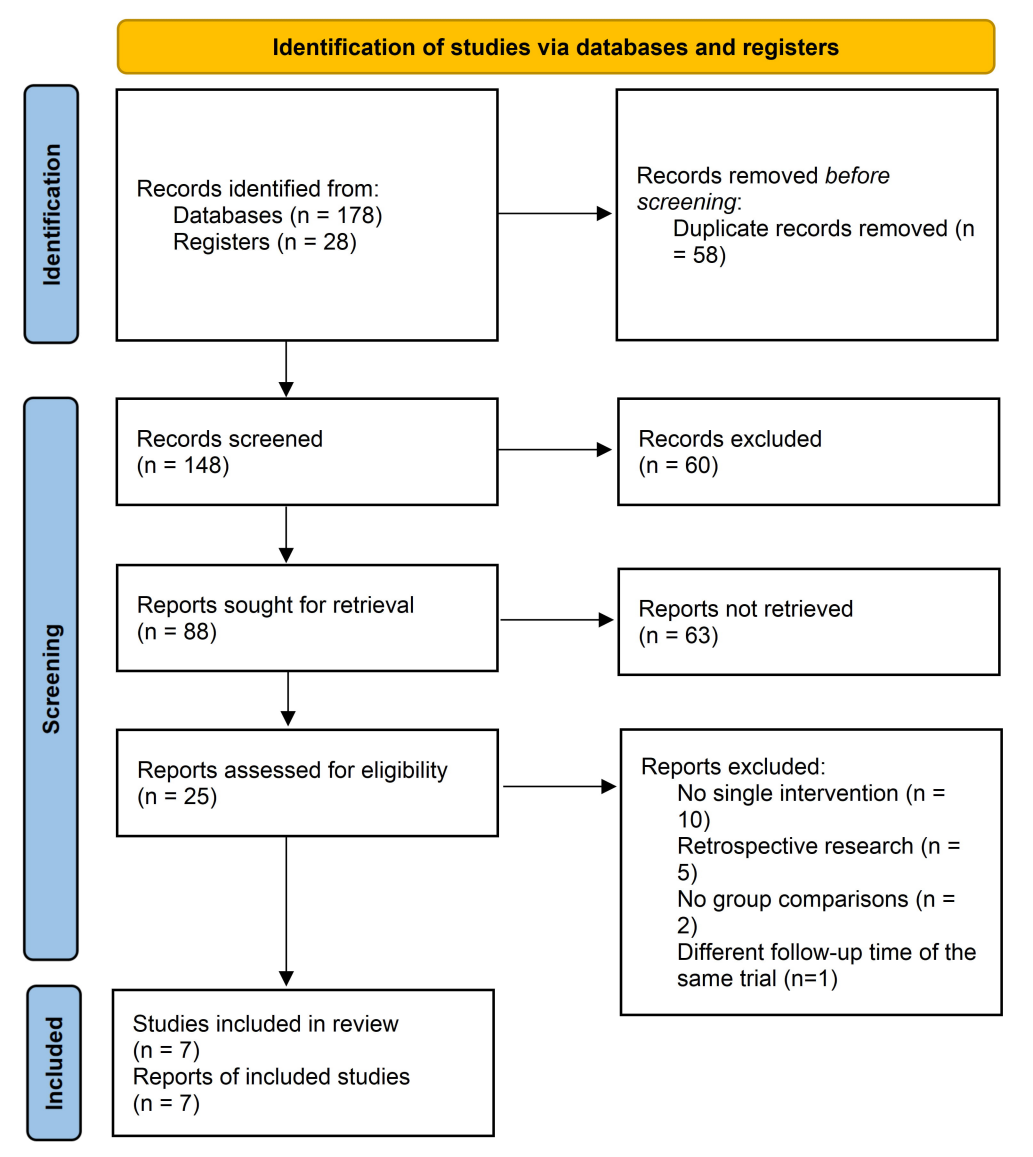
**Flow chart of literature search according the PRISMA statement**.

Our database search identified a total of 206 records, of which 58 were removed as duplicates. The titles and abstracts of the remaining 148 records resulted in the exclusion of 123 records for lacking relevance, being of a review study design, and being animal studies. The full-text review of the remaining 25 publications led to the exclusion of 10 articles for applying multiple interventions, five for using a retrospective study design, and two for lacking a comparison group. Thus, one record and six articles of seven prospective controlled trials were ultimately included in this meta-analysis [11, 12, 13, 14, 15, 16, 17]. The detailed study selection procedure is shown in the flow diagram (Fig. 1).

### 3.2 Risk of Bias

We used the Cochrane risk of bias tool to assess the bias risk of the seven included studies; the results are summarized in **Supplementary Figs. 1,2** in the Supplementary Materials. All included studies were prospective controlled trials, while the SFA ISR trial only reported brief results on ClinicalTrials.gov with no publications to date. Among the seven studies, the DEBATE-ISR trial contributed most to the unclear bias risk, as the researchers did not mention or describe their random methods used for sequence and allocation or their blinding methods in the intervention and outcome assessment. The ISAR-PEBIS trial failed to provide sufficient information regarding participant and personnel blinding. The COPA CABANA trial failed to present sufficient data as claimed in the methods. The SFA ISR trial was definitely sponsored by industry, although the researchers claimed that the sponsor did not employ principal investigators. Only three trials [12, 13, 15] met all standards of the Cochrane risk of bias tool and achieved a low risk of bias. All studies except the COPA CABANA trial reported outcomes completely as declared in their methods sections. Losses to follow-up were reported by all trials. An intention-to-treat analysis was used in all trials. Only two trials [14, 16] claimed no conflicts of interest, whereas four trials [12, 14, 16, 17] reported their sponsorship and the others did not mention funding.

### 3.3 Baseline Characteristics

The main demographic and clinical features of the seven included trials are shown in Table 1. The primary and secondary outcomes of the selected studies are presented in Table 2, and data that were eventually analyzed had to be provided by at least three studies. The seven trials [11, 12, 13, 14, 15, 16, 17] all started between 2010 and 2016, and five trials [11, 12, 13, 14, 16] performed in Europe started in 2010 and 2011.

**Table 1.** **Main features of included trials**.

		DEBATE-ISR		FAIR		PACUBA		ISAR-PEBIS		Liao		COPA CABANA		SFA ISR	
		PCB (n = 44)	UCB (n = 42)	PCB (n = 62)	UCB (n = 57)	PCB (n = 35)	UCB (n = 39)	PCB (n = 36)	UCB (n = 34)	PCB (n = 38)	UCB (n = 36)	PCB (n = 47)	UCB (n = 41)	PCB (n = 53)	UCB (n = 29)
Country		Italy		Germany		Austria		Germany		China		Germany		USA	
Year		2010–2011		2010–2012		2010–2012		2010–2013		2016–2018		2011–2013		2014–2017	
Multi-center		No		Yes		Yes		Yes		No		Yes		Yes	
Age, y		74 ± 11	76 ± 7	69 ± 8	67 ± 9	68.1 ± 9.2	68.3 ± 0.4	70 ± 10	68 ± 10	66.9 ± 9.0	67.2 ± 8.6	68.3 ± 9.6	67.6 ± 10.2	68.9 ± 9.35	67.0 ± 8.64
Male gender		32 (72.7%)	23 (54.8%)	33 (53.2%)	49 (70.2%)	20 (57%)	23 (59%)	24 (67%)	24 (70%)	22 (57.9%)	18 (50%)	26 (55%)	26 (63%)	30 (56.5%)	12 (41.4%)
Diabetes mellitus		44 (100%)	42 (100%)	28 (45.2%)	17 (29.8%)	17 (52%)	13 (38%)	12 (33%)	12 (35%)	19 (50%)	17 (47.2%)	20 (43%)	19 (46%)	NA	NA
Hypertension		39 (88.6%)	38 (90.5%)	52 (83.9%)	53 (93%)	26 (79%)	27 (79%)	33 (92%)	30 (88%)	30 (78.9%)	28 (77.8%)	38 (81%)	30 (73%)	NA	NA
CAD		9 (20.5%)	12 (28.6%)	26 (41.9%)	22 (38.6%)	12 (36%)	14 (41%)	17 (47%)	16 (47%)	13 (34.2%)	13 (36.1%)	10 (21%)	10 (24%)	NA	NA
Smoking		14 (31.8%)	11 (26.2%)	44 (70.9%)	46 (80.7%)	17 (52%)	18 (53%)	21 (58%)	24 (71%)	18 (47.4%)	16 (44.4%)	14 (30%)	15 (37%)	NA	NA
Obesity (BMI ≥30 ${\mathrm{kg/m}}^{2}$)		NA	NA	12 (19.4%)	12 (21.1%)	7 (22%)	7 (21%)	27 ± 4 ^a^	28 ± 4 ^a^	2 (5.3%)	1 (2.8%)	NA	NA	NA	NA
CKD		1.39 ± 1.5 ^b^	1.42 ± 1.5 ^b^	8 (12.9%)	10 (17.5%)	6 (19%)	6 (16%)	73 ± 33 ^c^	80 ± 23 ^c^	NA	NA	NA	NA	NA	NA
ABI		0.32 ± 0.11	0.36 ± 0.9	0.63 ± 0.27	0.64 ± 0.25	0.65 ± 0.16	0.65 ± 0.16	0.6 ± 0.3	0.7 ± 0.2	0.50 ± 0.13	0.52 ± 0.13	0.72 ± 0.23	0.65 ± 0.25	NA	NA
Rutherford class														NA	NA
	2			27 (43.5%)	27 (47.4%)	3 (9%)	8 (21%)	1 (3%)	0 (0%)	7 (18.4%)	8 (22.2%)	8 (17%)	3 (8%)	NA	NA
	3	11 (25%) ^d^	14 (33.3%) ^d^	32 (51.6%)	24 (42.1%)	32 (91%)	30 (79%)	34 (94%)	33 (97%)	14 (36.8%)	15 (41.7%)	36 (77%)	30 (79%)	NA	NA
	4			1 (1.6%)	6 (10.5%)	0 (0%)	0 (0%)	0 (0%)	0 (0%)	15 (39.5%)	11 (30.5%)	1 (2%)	2 (5%)	NA	NA
	5	33 (75%) ^d^	28 (66.7%) ^d^	2 (3.2%)	0 (0%)	0 (0%)	0 (0%)	1 (3%)	1 (3%)	2 (5.3%)	2 (5.6%)	2 (4%)	3 (8%)	NA	NA
	6			0 (0%)	0 (0%)	0 (0%)	0 (0%)	0 (0%)	0 (0%)	0 (0%)	0 (0%)	0 (0%)	0 (0%)	NA	NA
ISR Tosaka classification															
	I	7 (15%)	6 (16%)	16 (25.8%)	16 (28.1%)	8 (23%)	2 (5%)	10 (28%)	17 (50%)	9 (23.7%)	6 (16.7%)	NA	NA	NA	NA
	II	15 (34%)	8 (19%)	32 (51.6%)	30 (52.6%)	16 (46%)	26 (67%)	13 (36%)	7 (21%)	13 (34.2%)	15 (41.7%)	NA	NA	NA	NA
	III	22 (51%)	28 (65%)	14 (22.6%)	11 (19.3%)	11 (31%)	11 (28%)	13 (36%)	10 (29%)	16 (42.1%)	15 (41.7%)	NA	NA	NA	NA
Lesion length, mm		132 ± 86	137 ± 82	82.3 ± 70.9	81.1 ± 66.2	173 ± 113	184 ± 88	132 ± 65	146 ± 69	179 ± 80	182 ± 91	152 ± 85	128 ± 84	NA	NA
RVD, mm		4.9 ± 0.4	5.0 ± 0.5	5.1 ± 0.9	5.4 ± 0.5	5.7 ± 1.0	5.4 ± 0.9	4.8 ± 1.3	4.8 ± 1.2	5.9 ± 0.7	6.0 ± 0.9	5.2 ± 0.6	5.1 ± 0.8	NA	NA
MLD, mm		0.4 ± 0.5	0.3 ± 0.4	NA	NA	NA	NA	1 ± 0.8	0.8 ± 0.7	NA	NA	NA	NA	NA	NA
Diameter stenosis, %		91.3 ± 9.7	93.8 ± 9.0	89.0 ± 8.9	89.9 ± 9.6	NA	NA	80 ± 16	80 ± 16	90.2 ± 9.8	89.9 ± 9.6	91.4 ± 9.0	92.0 ± 9.1	NA	NA
Inclusion criteria		Diabetic patients with femoropopliteal ISR.		SFA ISR of up to 20 cm in length; diameter stenosis ≥70%; the popliteal artery and one of the infrapopliteal vessels had to be patent for sustained distal runoff; Rutherford category 2 to 4.		Age >50; symptomatic PAD (Rutherford-Becker category 2 or 3); ISR >50% in the SFA and P1 segment of the popliteal artery; a distal runoff of at least one artery.		Symptomatic ISR >70% or occlusion of SFA at the stented site.		Age 18 to 85 years; Rutherford Class 2 to 5; BMS implantation time from six months to three years; ABI <0.9 in the target limb; significant ISR ≥50% diameter stenosis in the SFA and P1 segment of the popliteal artery, reference vessel diameter of 3 to 7 mm; at least one patent crural runoff vessel to the foot.		ISR ≥70% or in-stent occlusion 3 to 27 cm long within the stent and adjacent segments of the SFA and/or popliteal artery occurring >3 months after stent implantation; Rutherford category 2 to 5 ischemia; at least one patent runoff vessel; patient willingness and ability to continue study participation.		Age above 18 years; Rutherford Category 2–4; significant (≥50%) restenosis of a previous bare nitinol stent in the femoropopliteal artery; lesion between 4 and 18 cm; target vessel diameter between 4 and 6 mm and meet device size; a patent free inflow artery; successful access for a guidewire; at least one patent native outflow artery to the ankle; no other prior vascular or surgical interventions recently.	
Exclusion criteria		Paclitaxel allergy; contraindication to combined antiplatelet treatment; life expectancy <1 year.		Untreated ipsilateral iliac artery stenosis; ongoing dialysis treatment; treatment with oral anticoagulants other than antiplatelet agents.		Inability to give written informed consent; known allergy, hypersensitivity, or intolerance to radiologic contrast media, aspirin, clopidogrel or ticlopidine, and paclitaxel; creatinine >2.5 mg/dL.		Acute ischemia and/or acute thrombosis of the SFA; untreated ipsilateral iliac or popliteal artery stenosis >70%; severe renal insufficiency; life expectancy <1 year; any contraindication to study medications.		Untreated ipsilateral iliac artery stenosis; ongoing dialysis treatment; aneurysm within target lesion; known intolerance or allergy to aspirin, heparin, clopidogrel, paclitaxel; planned amputation of the target limb; life expectancy <1 year.		No patent distal runoff vessel; guidewire unable to arrive the ISR lesion; presence of stent fracture grades 2 to 4; persistent inflow lesion, acute thrombosis of the study lesion; planned major amputation; aneurysm in the target vessel; abnormal platelet count or leukopenia; known intolerance or allergy to paclitaxel or any anticoagulation or antiplatelet agent.		Life expectancy of <1 year; patient is occupied by other researches; history of stroke within three months; history of MI, thrombolysis or angina within two weeks of enrollment; prior vascular surgery of the index limb; target lesion involves a previously placed covered stent or drug-eluting stent; grade 4 or 5 stent fracture in the restenotic stent; inability to take required study medications or allergy to contrast; known inadequate distal outflow or planned future treatment of vascular disease distal to the target lesion; intended use of adjunctive treatment modalities.	
Intervention		PEBA	UCBA	PEBA	UCBA	PEBA	UCBA	PEBA	UCBA	PCBA	UCBA	PCBA	UCBA	PCBA	UCBA
Follow-up/time		Yes/1, 6, 12, 24 and 36 months		Yes/6 and 12 months		Yes/24 hours, 1, 6 and 12 months		Yes/6, 8 and 24 months		Yes/1, 6 and 12 months		Yes/6, 12 and 24 months		Yes/1, 6 and 12 months	
Primary endpoints		Binary recurrent restenosis/incidence of TLR		Binary recurrent restenosis		Primary patency		Percentage diameter stenosis		Primary patency		Late lumen loss		Primary patency	
Secondary endpoints		Incidence of clinical-driven TLR; MAEs.		Primary angiographic success; freedom from TLR; ABI; clinical improvement ^e^; MAEs.		Technical success; clinical improvement; ABI; clinically driven TLR; MAEs.		Binary recurrent restenosis; freedom from TLR; MAEs.		Clinical-driven TLR; ABI; MAEs; walking impairment questionnaire (WIQ); quality of life measures; 6-minute walking test.		Binary restenosis; restenosis pattern; freedom from TLR; clinical improvement; ABI; MAEs.		Technical success; procedural success; secondary patency; freedom from TLR; clinical improvement; ABI; quality of life; MAEs.	
Post-procedure antiplatelet therapy		Dual-antiplatelet therapy (aspirin 100 mg/d plus clopidogrel 75 mg/d) for at least four weeks, then only aspirin was continued indefinitely.		Dual-antiplatelet therapy (aspirin 100 mg/d indefinitely plus clopidogrel 75 mg/d) for at least six month.		Dual-antiplatelet therapy (aspirin 100 mg/day indefinitely and clopidogrel 75 mg/day) for three months.		Dual-antiplatelet therapy (aspirin 100 mg per day indefinitely and clopidogrel 75 mg per day) for at least six months.		Dual-antiplatelet therapy (aspirin 100 mg/day indefinitely and clopidogrel 75 mg/day) for three months, then only aspirin was continued.		Dual-antiplatelet therapy (aspirin 100 mg/d plus clopidogrel 75 mg/d) for at least four weeks, then only aspirin was continued as a lifelong therapy.		NA	
Registration no.		NCT01558531		NCT01305070		NCT01247402		NCT01083394		ChiCTR1800017055		NCT01594684		NCT02063672	

Continuous data are presented as the means ± standard deviation; dichotomous data are given as the counts (percentage). DEBATE-ISR, Drug-Eluting Balloon in Peripheral Intervention for In-Stent Restenosis trial; FAIR, Femoral Artery In-Stent Restenosis trial; PACUBA, Paclitaxel Balloon Versus Standard Balloon in In-Stent Restenosis of the Superficial Femoral Artery trial; ISAR-PEBIS, Paclitaxel-Eluting Balloon Versus Conventional Balloon Angioplasty for In-Stent Restenosis of Superficial Femoral Artery trial; Liao, Orchid Drug-Coated Balloon Versus Standard Percutaneous Transluminal Angioplasty for Treatment of Femoropopliteal Artery In-Stent Restenosis trial; COPA CABANA, Cotavance Paclitaxel-Coated Balloon versus Uncoated Balloon Angioplasty for Treatment of In-Stent Restenosis in SFA and the Popliteal Artery; SFA ISR, Lutonix® Drug Coated Balloon versus Standard Balloon Angioplasty for Treatment of Femoropopliteal In-Stent Restenosis trial; PCB, paclitaxel-coated balloon; UCB, uncoated balloon; CAD, coronary artery disease; BMI, body mass index; CKD, chronic kidney disease; ABI, ankle-brachial index; ISR, in-stent restenosis; RVD, reference vessel diameter; MLD, minimal lumen diameter; SFA, superficial femoral artery; BMS, bare-metal stent; PEBA, paclitaxel-eluting balloon angioplasty; UCBA, uncoated balloon angioplasty; PCBA, paclitaxel-coated balloon; TLR, target lesion revascularization; MAEs, major adverse events; NA, not applicable. a: BMI, ${\mathrm{kg/m}}^{2}$. b: Serum creatinine, mg/dL. c: Glomerular filtration rate, mL/min. d: The authors only give numbers of patient with Rutherford category ≥4. e: Clinical improvement of ≥1 Rutherford category.

**Table 2.** **Primary and secondary outcomes**.

	DEBATE-ISR		FAIR		PACUBA		ISAR-PEBIS		Liao		COPA CABANA		SFA ISR		Total	
	PCB	UCB	PCB	UCB	PCB	UCB	PCB	UCB	PCB	UCB	PCB	UCB	PCB	UCB	PCB	UCB
Recurrent restenosis (6 months)	NA	NA	8/52 (15.4%)	21/47 (44.7%)	13/33 (39.4%)	21/31 (67.7%)	8/27 (29.6%)	16/27 (59.3%)	NA	NA	5/37 (13.5%)	16/27 (59.3%)	NA	NA	34/149 (22.8%)	74/132 (56.1%)
Recurrent restenosis (12 months)	8/44 (18.2%)	28/42 (66.7%)	13/44 (29.5%)	25/40 (62.5%)	17/26 (65.4%)	25/28 (89.3%)	NA	NA	NA	NA	NA	NA	NA	NA	38/114 (33.3%)	78/110 (70.9%)
Primary patency (12 months)	NA	NA	NA	NA	5/13 (38.5%)	1/7 (14.3%)	NA	NA	29/33 (87.9%)	16/31 (51.6%)	NA	NA	33/49 (67.3%)	11/23 (47.8%)	67/95 (70.5%)	28/61 (45.9%)
Freedom from TLR (6 months)	NA	NA	51/53 (96.2%)	36/45 (80.0%)	30/33 (90.1%)	26/31 (83.9%)	31/36 (86.1%)	26/34 (76.5%)	NA	NA	44/45 (97.8%)	34/38 (89.5%)	47/50 (94.0%)	24/26 (92.3%)	203/217 (93.5%)	146/174 (83.9%)
Freedom from TLR (12 months)	38/44 (86.4%)	29/42 (69.0%)	37/41 (90.2%)	13/25 (52.0%)	8/16 (50.0%)	4/18 (22.2%)	26/33 (78.8%)	20/33 (60.6%)	31/33 (93.9%)	20/31 (64.5%)	37/43 (86.0%)	19/37 (51.4%)	34/43 (79.1%)	10/16 (62.5%)	211/258 (83.4%)	115/202 (56.9%)
Clinical improvement ^a^ (6 months)	NA	NA	36/51 (70.6%)	27/47 (57.4%)	20/26 (76.9%)	14/25 (56.0%)	NA	NA	NA	NA	7/29 (24.1%)	4/27 (14.8%)	34/49 (69.4%)	13/22 (59.1%)	97/155 (62.6%)	58/121 (47.9%)
Clinical improvement ^a^ (12 months)	34/44 (77.3%)	25/42 (59.5%)	35/45 (77.8%)	23/44 (52.3%)	11/16 (68.8%)	6/11 (54.5%)	NA	NA	25/33 (75.8%)	16/31 (51.6%)	NA	NA	30/49 (61.2%)	11/22 (50.0%)	135/187 (72.2%)	81/150 (54.0%)
ABI (12 months)	NA	NA	0.86 ± 0.30	0.90 ± 0.17	0.79 ± 0.20	0.84 ± 0.30	NA	NA	0.82 ± 0.11	0.70 ± 0.15	NA	NA	NA	NA	NA	NA
MAEs (6 months)	NA	NA	1/55 (1.8%)	2/47 (4.3%)	NA	NA	1/36 (2.8%)	0/34 (0%)	NA	NA	NA	NA	5/52 (9.6%)	4/27 (14.8%)	7/143 (4.9%)	6/108 (5.6%)
MAEs (12 months)	7/44 (15.9%)	14/42 (33.3%)	4/47 (8.5%)	5/44 (11.4%)	NA	NA	NA	NA	1/38 (2.6%)	2/34 (5.9%)	NA	NA	10/49 (20.4%)	7/23 (30.4%)	22/178 (12.4%)	28/143 (19.6%)

Continuous data are presented as the means ± standard deviation; dichotomous data are given as the counts (percentage). DEBATE-ISR, Drug-Eluting Balloon in Peripheral Intervention for In-Stent Restenosis trial; FAIR, Femoral Artery In-Stent Restenosis trial; PACUBA, Paclitaxel Balloon Versus Standard Balloon in In-Stent Restenosis of the Superficial Femoral Artery trial; ISAR-PEBIS, Paclitaxel-Eluting Balloon Versus Conventional Balloon Angioplasty for In-Stent Restenosis of Superficial Femoral Artery trial; Liao, Orchid Drug-Coated Balloon Versus Standard Percutaneous Transluminal Angioplasty for Treatment of Femoropopliteal Artery In-Stent Restenosis trial; COPA CABANA, Cotavance Paclitaxel-Coated Balloon versus Uncoated Balloon Angioplasty for Treatment of In-Stent Restenosis in SFA and the Popliteal Artery; SFA ISR, Lutonix® Drug Coated Balloon versus Standard Balloon Angioplasty for Treatment of Femoropopliteal In-Stent Restenosis trial; PCB, paclitaxel-coated balloon; UCB, uncoated balloon; TLR, target lesion revascularization; ABI, ankle-brachial index; MAEs, major adverse events; NA, not applicable. a: Clinical improvement of ≥1 Rutherford category.

A total of 593 patients were enrolled in our analysis: 315 (53.1%) treated with PCBA and 278 (46.9%) treated with UCBA. All included studies were prospective controlled trials; of them, five [12, 13, 14, 16, 17] were multi-center. The FAIR trial enrolled 119 (20.1%) participants, and the numbers in the other trials were approximately the same. Patients were allocated to two groups in a 1:1 ratio except in the SFA ISR trial. The enrolled patients were mainly male, elderly, and those with CAD risk factors such as smoking, hypertension, DM, and CKD. On account of the trial objective, participants in the DEBATE-ISR trial all had DM. The dominant inclusion criteria were Rutherford categories 2–5, FP ISR, and at least one distal runoff. The primary outcomes were not completely the same but they were all related to the assessment of target lesion restenosis.

In addition to the comparison among the baseline characteristics of the seven trials, the features of every trial were also important. The features of the PCB and UCB groups in each trial were similar. The intervention strategies of the seven trials were the same referring to PCBA for the PCB group and UCBA for the UCB group.

### 3.4 Outcomes

The meta-analysis outcomes of the entire study are summarized in Table 3. None of the outcomes showed any publication bias. We calculated pooled ORs and WMDs for the dichotomous and continuous variables.

**Table 3.** **Summary meta-analysis outcomes in the entire study**.

Outcome measure	Number of studies	Meta-analysis model	OR (95% CI)	*p* value	Publication bias (*p*)
Recurrent restenosis (6 months)	4	Fixed effects	0.22 (0.13–0.38)	<0.00001	0.446
Recurrent restenosis (12 months)	3	Fixed effects	0.18 (0.10–0.33)	<0.00001	0.961
Primary patency (12 months)	3	Fixed effects	3.59 (1.72–7.47)	0.0006	0.743
Freedom from TLR (6 months)	5	Fixed effects	2.70 (1.36–5.35)	0.005	0.793
Freedom from TLR (12 months)	7	Fixed effects	4.02(2.55–6.34)	<0.00001	0.213
Clinical improvement ^a^ (6 months)	4	Fixed effects	1.87 (1.10–3.16)	0.02	0.624
Clinical improvement ^a^ (12 months)	5	Fixed effects	2.38 (1.50–3.79)	0.0002	0.582
ABI (12 months)	3	Random effects	0.02 (–0.11–0.14)^b^	0.77	0.285
MAEs (6 months)	3	Fixed effects	0.71 (0.24–2.14)	0.54	0.608
MAEs (12 months)	4	Fixed effects	0.50 (0.27–0.96)	0.04	0.717

TLR, target lesion revascularization; ABI, ankle-brachial index; MAEs, major adverse events; OR, odds ratio; CI, confidence interval. a: Clinical improvement of ≥1 Rutherford category. b: Weighted mean difference.

#### 3.4.1 Recurrent Restenosis

**Fig. 2.**
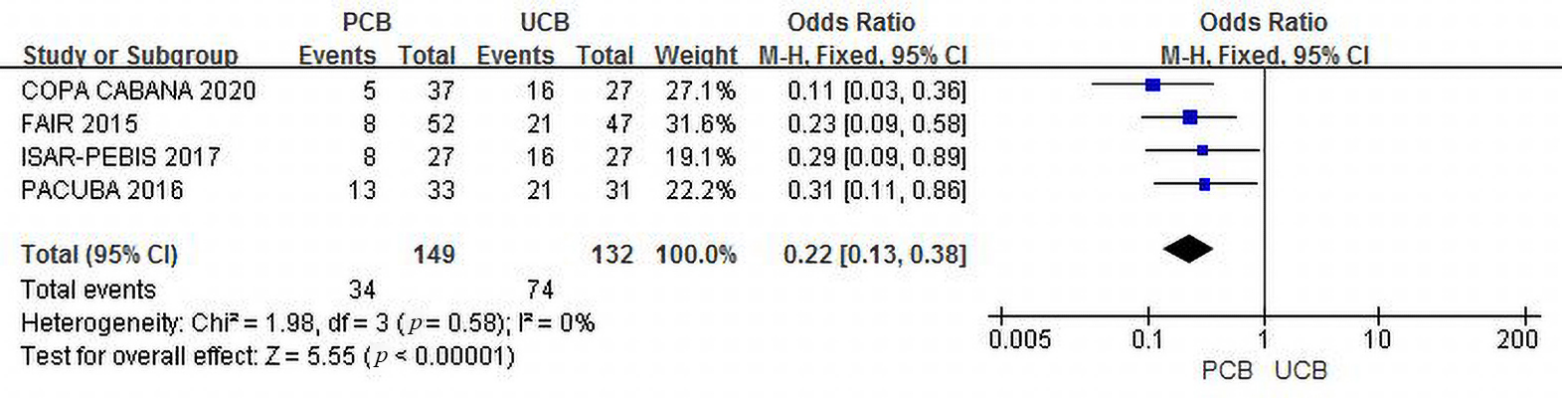
**Forest plot of estimated individual and overall effect of recurrent restenosis between PCB and UCB groups at 6 months**.

Four trials [12, 13, 14, 16] evaluated recurrent restenosis at 6 months of follow-up. The incidence of combined recurrent restenosis was significantly lower in the PCB group than in the UCB group (22.8% and 56.1%, respectively). The summary OR was 0.22 (95% CI, 0.13–0.38; Z = 5.55; *p *< 0.00001). There was no heterogeneity across the four trials ($\chi^{2}$ = 1.98; *p* = 0.58; $I^{2}$ = 0%) (Fig. 2).

**Fig. 3.**
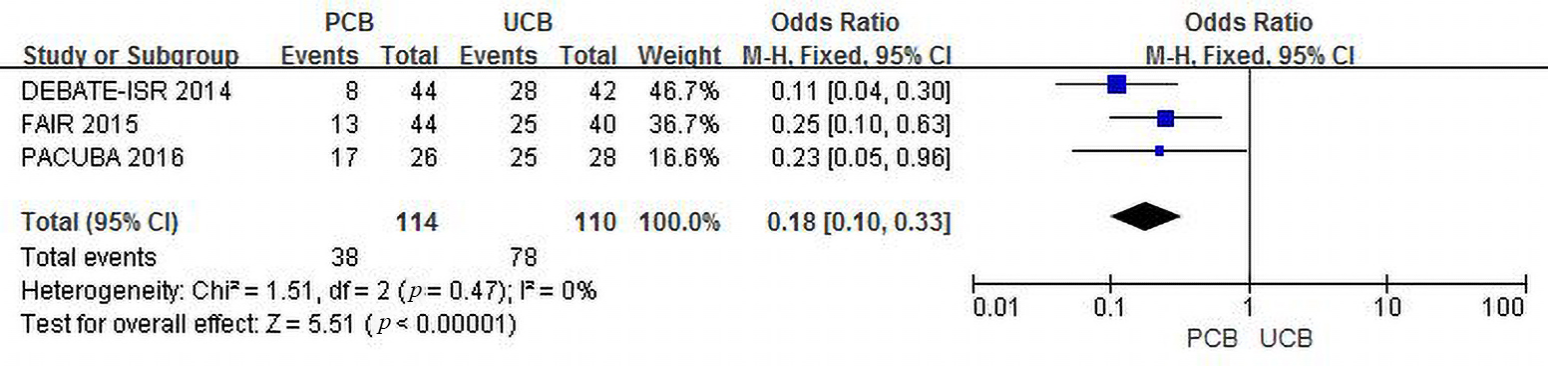
**Forest plot of estimated individual and overall effect of recurrent restenosis between PCB and UCB groups at 12 months**.

Three trials [11, 12, 13] evaluated recurrent restenosis at the 12-month follow-up. Although the recurrent restenosis rate in both groups increased, the incidence of combined recurrent restenosis in the PCB group was significantly lower than that in the UCB group (33.3% versus 70.9%, respectively). The summary OR was 0.18 (95% CI, 0.10–0.33; Z = 5.51; *p *< 0.00001). There was no heterogeneity across the three trials ($\chi^{2}$ = 1.51; *p* = 0.47; $I^{2}$ = 0%) (Fig. 3).

Funnel plots of both outcomes are shown in the Supplementary Materials (**Supplementary Figs. 3,4**).

#### 3.4.2 Primary Patency

**Fig. 4.**
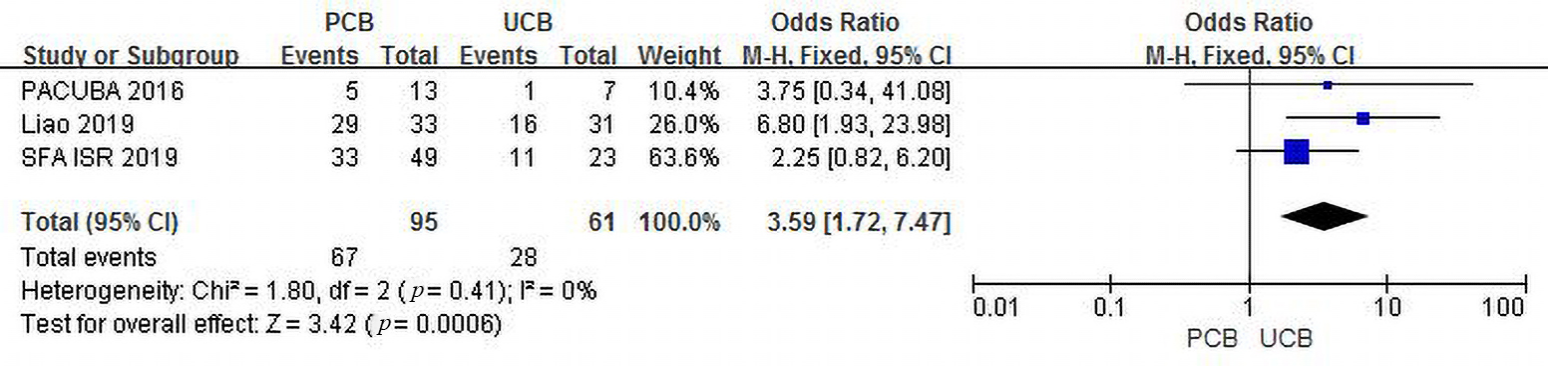
**Forest plot of estimated individual and overall effect of primary patency between PCB and UCB groups at 12 months**.

Three trials [13, 15, 17] evaluated primary patency at 12 months for the PCB and UCB groups. The incidence of combined primary patency was 70.5% in the PCB group and 45.9% in the UCB group, and the summary OR was 3.59 (95% CI, 1.72–7.47; Z = 3.42; *p* = 0.0006), reflecting a significant intergroup difference. There was no heterogeneity across trials ($\chi^{2}$ = 1.80; *p* = 0.41; $I^{2}$ = 0%) (Fig. 4). The funnel plot of this outcome has been added to the Supplementary Materials (**Supplementary Fig. 5**).

#### 3.4.3 Freedom from TLR

**Fig. 5.**
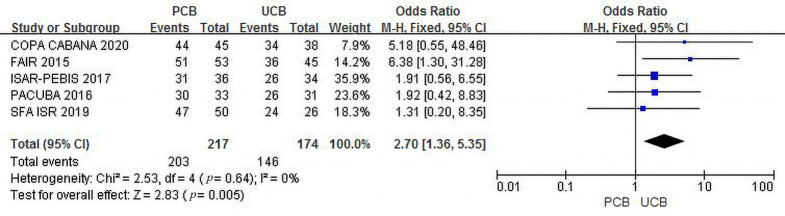
**Forest plot of estimated individual and overall effect of freedom from TLR between PCB and UCB groups at 6 months**.

Five trials [12, 13, 14, 16, 17] evaluated freedom from TLR at the 6-month follow-up. The incidence of combined freedom from TLR was 93.5% in the PCB group versus 83.9% in the UCB group, and the summary OR was 2.70 (95% CI, 1.36–5.35; Z = 2.83; *p* = 0.005), indicating an existing intergroup difference. There was no heterogeneity across trials ($\chi^{2}$ = 2.53; *p* = 0.64; $I^{2}$ = 0%) (Fig. 5).

**Fig. 6.**
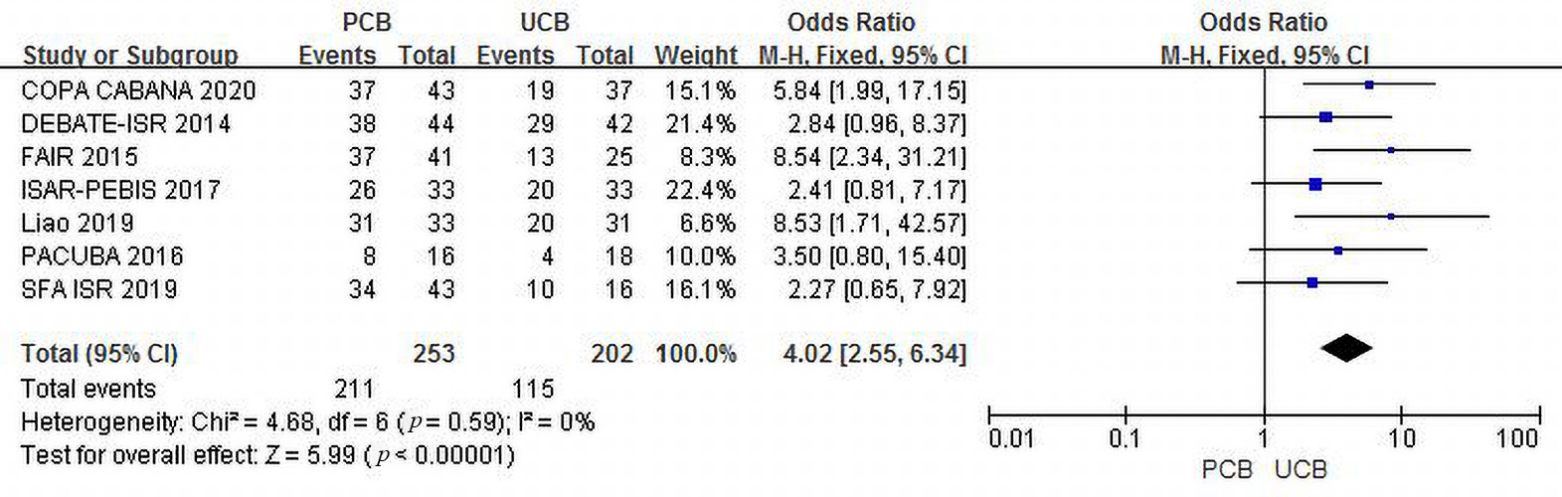
**Forest plot of estimated individual and overall effect of freedom from TLR between PCB and UCB groups at 12 months**.

All seven trials [11, 12, 13, 14, 15, 16, 17] evaluated freedom from TLR at the 12-month follow-up. The incidence of combined freedom from TLR in the two groups was 83.4% versus 56.9%, and the summary OR was 4.02 (95% CI, 2.55–6.34; Z = 5.99; *p *< 0.00001), which indicated a significant intergroup difference. There was no heterogeneity across trials ($\chi^{2}$ = 4.68; *p* = 0.59; $I^{2}$ = 0%) (Fig. 6).

Funnel plots of both outcomes are shown in the Supplementary Materials (**Supplementary Figs. 6,7**).

#### 3.4.4 Clinical Improvement

**Fig. 7.**
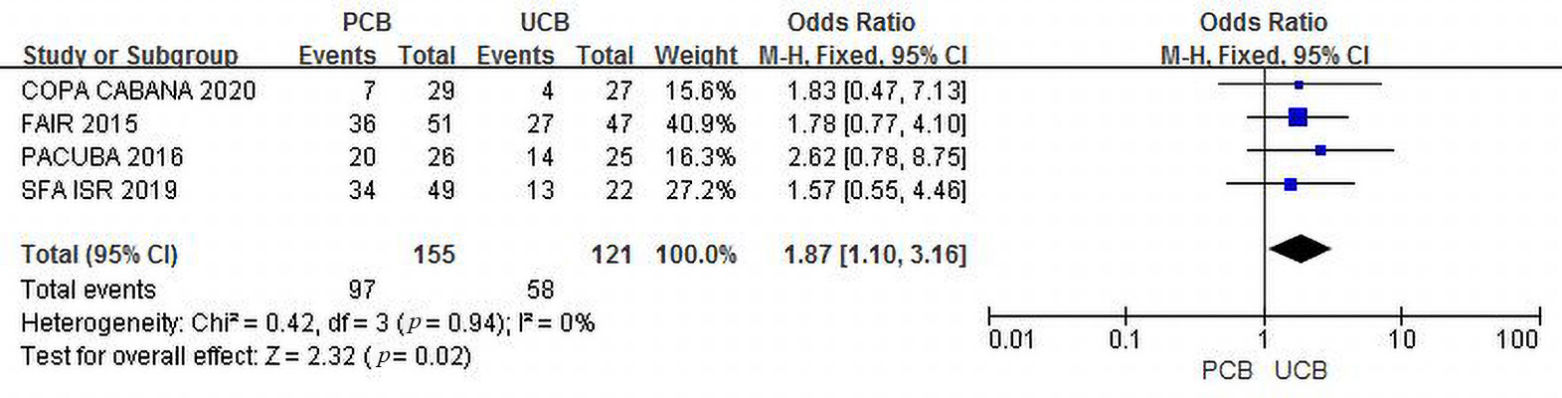
**Forest plot of estimated individual and overall effect of clinical improvement between PCB and UCB groups at 6 months**.

Four trials [12, 13, 16, 17] evaluated clinical improvement at 6 months for the PCB and UCB groups. The incidence of combined clinical improvement was significantly higher in the PCB group (62.6%) than in the UCB group (47.9%). The summary OR was 1.87 (95% CI, 1.10–3.16; Z = 2.32; *p* = 0.02). There was no heterogeneity across trials ($\chi^{2}$ = 0.42; *p* = 0.94; $I^{2}$ = 0%) (Fig. 7).

**Fig. 8.**
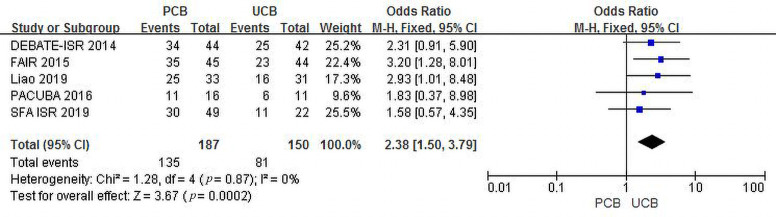
**Forest plot of estimated individual and overall effect of clinical improvement between PCB and UCB groups at 12 months**.

Five trials [11, 12, 13, 15, 17] evaluated clinical improvement at 12 months for the PCB and UCB groups. The incidence of combined clinical improvement in the PCB group (72.2%) was significantly higher than that in the UCB group (54.0%). The summary OR was 2.38 (95% CI, 1.50–3.79; Z = 3.67; *p* = 0.0002). There was no heterogeneity across trials ($\chi^{2}$ = 1.28; *p* = 0.87; $I^{2}$ = 0%) (Fig. 8).

Funnel plots of both outcomes are shown in the Supplementary Materials (**Supplementary Figs. 8,9**).

#### 3.4.5 Ankle-Brachial Index

**Fig. 9.**
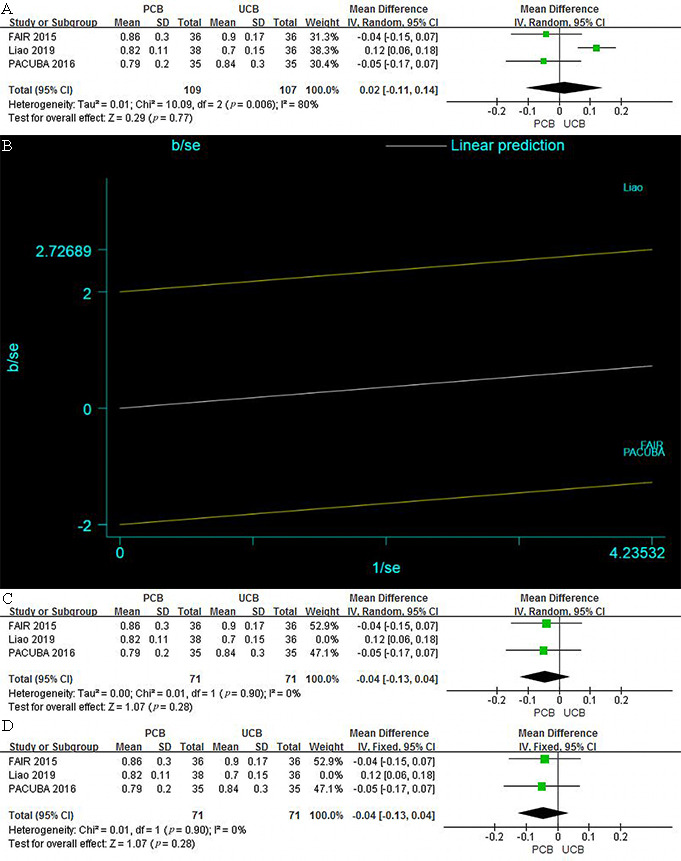
**Overall effect of the ABI between PCB and UCB groups at 12 months**. (A) Forest plot of estimated individual and overall effect of the ABI between PCB and UCB groups at 12 months before revised. (B) Galbraith plot of estimated individual and overall effect of the ABI between PCB and UCB groups at 12 months. (C) Revised forest plot of estimated individual and overall effect of the ABI between PCB and UCB groups at 12 months with random-effect model. (D) Revised forest plot of estimated individual and overall effect of the ABI between PCB and UCB groups at 12 months with fixed-effect model.

Three trials [12, 13, 15] evaluated ABI at 12 months for the PCB and UCB groups. No significant intergroup difference was noted, and the mean difference was 0.02 (95% CI, –0.11–0.14; Z = 0.29; *p* = 0.77). There was high heterogeneity across trials ($T^{2}$ = 0.01; $\chi^{2}$ = 10.09; *p* = 0.006; $I^{2}$ = 80%) (Fig. 9A).

Considering the existence of high heterogeneity, we performed a sensitivity analysis and created a Galbraith plot (Fig. 9B) and found that the heterogeneity came from the Liao trial. To confirm the source of the heterogeneity, we removed the Liao trial, recalculated the meta-analysis, and generated a new forest plot with both random- and fixed-effect models. As shown in Fig. 9C,D, the heterogeneity was thereby eliminated ($\chi^{2}$ = 0.01; *p* = 0.90; $I^{2}$ = 0%) with no difference between the two models, and the result was similar to that obtained before the revision. We then performed a regression analysis of these data with race, PTX dose, and balloon device as covariates. The *p* values were 0.818, 0.907, and 0.823, respectively, indicating that the heterogeneity was not due to any of these variables.

Funnel plot of the revised outcome is provided in the Supplementary Materials (**Supplementary Figs. 10**).

#### 3.4.6 Major Adverse Events

**Fig. 10.**
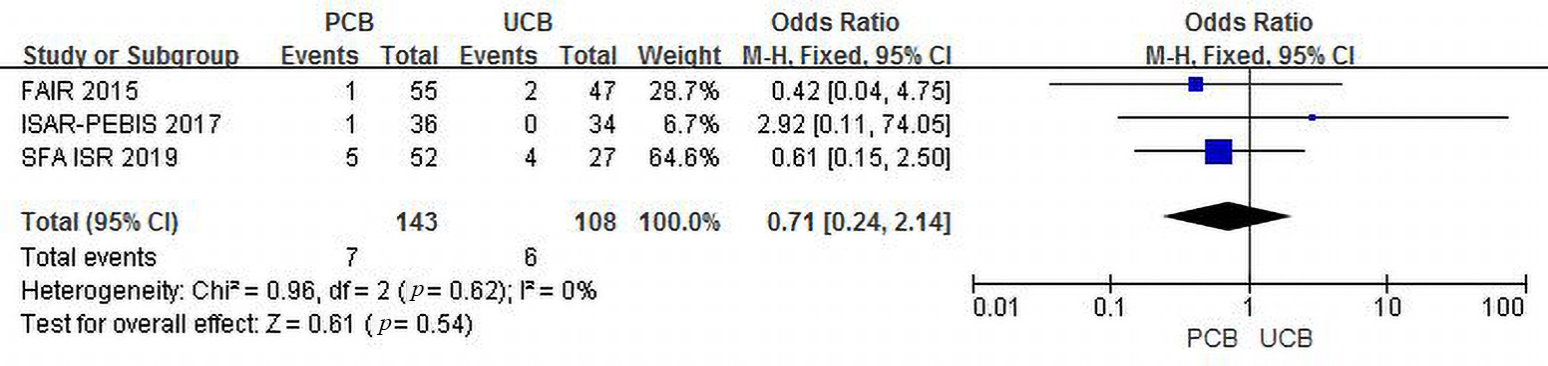
**Forest plot of estimated individual and overall effect of MAEs between PCB and UCB groups at 6 months**.

Three trials [12, 14, 17] evaluated MAEs at 6 months for the PCB and UCB groups. The incidence of combined MAEs was 4.9% and 5.6%, respectively. The summary OR was 0.71 (95% CI, 0.24–2.14; Z = 0.61; *p* = 0.54). There was no heterogeneity across trials ($\chi^{2}$ = 0.96; *p* = 0.62; $I^{2}$ = 0%) (Fig. 10).

**Fig. 11.**
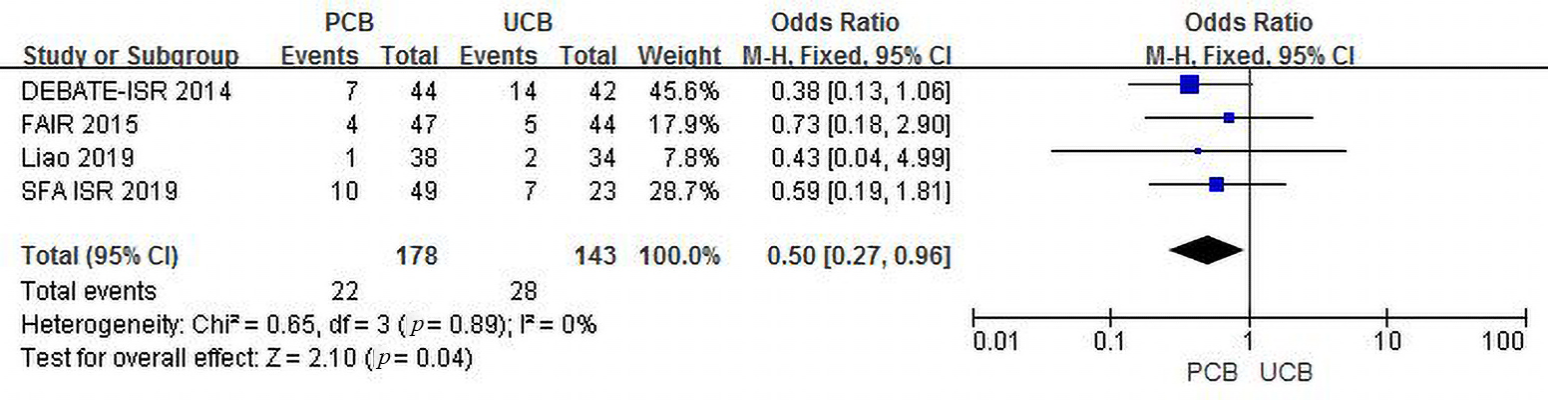
**Forest plot of estimated individual and overall effect of MAEs between PCB and UCB groups at 12 months**.

Four trials [11, 12, 15, 17] evaluated MAEs at 12 months for the PCB and UCB groups. The incidence of combined MAEs was 12.4% and 19.6%, respectively, and the summary OR of 0.50 (95% CI, 0.27–0.96; Z = 2.10; *p* = 0.04) indicated a statistically significant intergroup difference. There was no heterogeneity across trials ($\chi^{2}$ = 0.65; *p* = 0.89; $I^{2}$ = 0%) (Fig. 11).

Funnel plots of the outcomes are provided in the Supplementary Materials (**Supplementary Figs. 11,12**).

Considering that most patients in the trials [11, 12, 13, 14, 15, 16] continued dual-antiplatelet therapy after the procedure, adverse events probably resulting from the therapy should also be considered. The DEBATE-ISR study [11] reported an incidence of stroke at the 12-month follow-up of 2.3% in the PCB group versus 0% in the UCB group. The FAIR trial [12] calculated the cumulative incidence of major bleeding in patients at 6 and 12 months and reported 0% for both groups. The SFA ISR trial [17] provided a detailed record of various adverse events for up to 36 months. Among those reported, the cumulative incidence of ischemic and hemorrhagic accidents was 1.9% and 10.3% in the PCB and UCB groups, respectively.

### 3.5 Sensitivity Analysis

Sensitivity analyses were performed to determine the influence of a single study on the estimated overall effect. We performed the sensitivity analysis by recalculating the pooled estimates for recurrent restenosis, primary patency, freedom from TLR, clinical improvement, ABI, and MAEs and omitting one study at a time. Besides the positive result we reported in section 3.4.5 above with moderate to high heterogeneity, some other obvious results were found in the process.

As described in section 3.4.2, when the Liao trial was omitted from the calculation, the result changed (**Supplementary Fig. 13A,B**).

As described in section 3.4.3, when we omitted the FAIR trial and recalculated the meta-analysis, the results changed and indicated no significant difference between the PCB and UCB groups, which was opposite to our previous result (**Supplementary Fig. 13C,D**).

Similar conditions appeared in section 3.4.4. The results recalculated with the FAIR or PACUBA trial omitted both indicated no significant difference between the PCB and UCB groups (**Supplementary Fig. 13E,F**).

The last positive result was described in section 3.4.4, in which the recalculated results were different when we omitted the DEBATE-ISR or SFA ISR trial (**Supplementary Fig. 13G,H**).

### 3.6 Assessment of Evidence

The meta-analysis aimed to summarize and provide general evidence, so it was important to assess its quality. All six tables assessing the evidence of the different outcomes are listed in the Supplementary Materials (**Supplementary Tables 1–6**). A summary of these results is presented in Table 4. According to the assessment, most of the evidence was reliable. However, the outcome “clinical improvement (12 months)” (**Supplementary Table 4**) was of low quality, and the outcome “ABI (12 months)” (**Supplementary Table 5**) was of very low quality, which left a question about the two outcomes.

**Table 4.** **Summary of evidence level in the entire study**.

Outcome measure	Number of studies	Evidence level	Reasons
Recurrent restenosis (6 months)	4	High	None
Recurrent restenosis (12 months)	3	Moderate	Selection bias, performance bias and detection bias of DEBATE-ISR trial
Primary patency (12 months)	3	Moderate	Other bias (high missing follow-up rate) of PACUBA trial
Freedom from TLR (6 months)	5	Moderate	Result of FAIR trial is not consistent with others
Freedom from TLR (12 months)	7	Moderate	Selection bias, performance bias and detection bias of DEBATE-ISR trial
Clinical improvement (6 months)	4	High	None
Clinical improvement (12 months)	5	Low	(1) Selection bias, performance bias and detection bias of DEBATE-ISR trial; (2) Results of FAIR trial and Liao trial are not consistent with others
ABI (12 months)	3	Very low	(1) Attrition bias of COPA CABANA trial; (2) Result of Liao trial is not consistent with others
MAEs (6 months)	3	Moderate	Result of ISAR-PEBIS trial is not consistent with others
MAEs (12 months)	4	Moderate	Selection bias, performance bias and detection bias of DEBATE-ISR trial

DEBATE-ISR, Drug-Eluting Balloon in Peripheral Intervention for In-Stent Restenosis trial; FAIR, Femoral Artery In-Stent Restenosis trial; PACUBA, Paclitaxel Balloon Versus Standard Balloon in In-Stent Restenosis of the Superficial Femoral Artery trial; ISAR-PEBIS, Paclitaxel-Eluting Balloon Versus Conventional Balloon Angioplasty for In-Stent Restenosis of Superficial Femoral Artery trial; Liao, Orchid Drug-Coated Balloon Versus Standard Percutaneous Transluminal Angioplasty for Treatment of Femoropopliteal Artery In-Stent Restenosis trial; COPA CABANA, Cotavance Paclitaxel-Coated Balloon versus Uncoated Balloon Angioplasty for Treatment of In-Stent Restenosis in SFA and the Popliteal Artery; TLR, target lesion revascularization.

## 4. Discussion

Our results showed that, compared with UCBA, participants treated with PCBA experienced decreased recurrent restenosis, increased primary patency, freedom from TLR, and clinical improvement over a 1-year duration. However, the ABI and MAEs of patients did not differ significantly between the two interventions.

Guidelines recommend primary stenting as a class I intervention for FP lesions, but controversy persists regarding ISR. Although several endovascular therapies are available, they provide suboptimal long-term patency rates. Therefore, a few treatment methods are strongly recommended by these guidelines. In the 2018 Society for Cardiovascular Angiography and Interventions consensus guidelines for device selection in femoropopliteal arterial interventions [18], DCB was recommended as a class I treatment for managing FP ISR. In contrast, the 2017 ESC guidelines [3] provided a class IIb recommendation for DCB angioplasty for FP ISR. The evidence level of both guidelines was B, which partly explains this controversy. The quality of the evidence required improvement. Thus, it is important to confirm the efficacy and safety of PCBA versus UCBA.

Paclitaxel is currently a widely used drug in DES and DCB for its antiproliferative effect in vivo. To date, many types of DCB are available on the market [19]. Studies included in our analysis applied 5 different balloon products (IN.PACT Admiral, FREEWAY, Orchid, Cotavance and Lutonix), and the PTX dose of them is 3 and 3.5 μ${\mathrm{g/mm}}^{2}$. For the time being, the paclitaxel dose varies between 2 and 3.5 μ${\mathrm{g/mm}}^{2}$ [20], which contains the dose used in studies included. Another difference is the technology used in different products. However, only a few studies have compared different PCB directly, and made opposite conclusions [21, 22]. Thus, the present data are underpowered to discern outcome differences between the different PTX devices. In our analysis, there is no heterogeneity across trials in most outcomes, and the outcomes observe a similar trend, indicating that the devices may not influence the overall effects.

As shown in Figs. 2,3,4, our trial yielded reliable and consistent findings that support the benefits of PCBA. In brief, the recurrent restenosis and primary patency results revealed a better angiographic endpoint for PCBA versus UCBA. The outcomes reflected the vessel patency after the interventions measured on DUS and CTA as recommended by the guidelines [3]. Angiographic success laid the foundation for PCB preference in the management of FP ISR.

In our analysis, we chose freedom from TLR and clinical improvement as the clinical success parameters. Freedom from TLR was clinically driven and not based on imaging features. Clinical improvement was evaluated as an increase in Rutherford classification, a method related to clinical symptoms. As reported in Figs. 5,6,7,8, both outcomes supported that the clinical results of PCBA were superior to those of UCBA at one year post-intervention.

In our analysis, ABI did not differ significantly between PCBA and UCBA, but there was high heterogeneity across the three trials [12, 13, 15]. We confirmed the Liao trial as the source of the heterogeneity in the sensitivity analysis but failed to identify the variable causing the heterogeneity. The regression results reflected that race, PTX dose, and balloon device were not sources of heterogeneity. ABI was defined as the ratio of systolic blood pressure (SBP) measured at the ankle to that measured at the brachial artery, and it has become a good non-invasive test for diagnosing LEAD because of its good sensitivity and specificity [3]. In addition to its frequently used diagnostic function, ABI can be used as a follow-up parameter when combined with angiographic methods such as DUS for revascularized patients with PAD [23]. ABI was stable in most situations, but its sensitivity was poorer in patients with DM or end-stage CKD due to medial artery calcification (MAC) [24, 25]. As shown in Table 1, the FAIR and PACUBA trials reported a similar baseline ABI of approximately 0.65, while the Liao trial reported an ABI of approximately 0.50, and there was no significant difference between the PCB and UCB groups in terms of ABI. However, the proportion of patients with DM differed among the three trials. In the FAIR trial, the ratio was 45.2% in the PCB group and 29.8% in the UCB group. In the PACUBA trial, the rates were 52% and 38%, respectively. Only in the Liao trial were the ratios close between the two groups (50% versus 47.2%). We hypothesized that the existence of DM might explain the heterogeneity across the three trials, especially between the Liao trial and the other two trials. On the other hand, compared with the baseline values, the summarized results of the three trials [12, 13, 15] showed an approximate 0.20 increase in ABI at 12 months post-intervention, which was close to the threshold of 0.90 for diagnosing PAD [26]. The SFA ISR trial reported a similar increase in ABI. To make the calculation of ABI more precise, future researchers should maintain the consistency of baseline characteristics (DM, hypertension, smoking, CKD) between patients in the experimental and control groups. The toe-brachial index (TBI), another measurement when ABI is unsuitable, is generally unaffected by MAC with better sensitivity but lower specificity than ABI [23, 27].

As shown in Figs. 10,11, we can conclude that PCBA has a safety profile similar to that of UCBA. PCBA did not significantly increase the incidence of MAEs compared to UCBA. In contrast, PCBA significantly decreased the incidence of MAEs compared with UCBA 12 months post-intervention. UCBA has been the most commonly used strategy for PAD for a long time, and its side effects are relatively low [28]. Therefore, with a low side effect ratio, PCBA can also be safely applied to manage FP ISR. Besides, it is noteworthy that, the results of a meta-analysis published in 2018 aroused concern about an increased risk of death associated with the use of PCB to manage PAD [29]. Right after that, several researches [30, 31, 32] made a different conclusion that PCB was safe. Although the researchers did not reach an agreement, they all admitted that more data were needed, which made the safety of PCB still under controversy. In our analysis, there is no difference between PCBA and UCBA in the incidence of MAEs.

As illustrated in the sensitivity analysis, some outcomes were insufficiently stable, as characterized by a change in conclusion when one trial was omitted. Theoretically, a larger number of participants and a shorter 95% CI line in the forest plot represented a more reliable conclusion. In terms of the outcomes “clinical improvement at 6-month follow-up” and “MAEs at 12-month follow-up”, a relatively reliable trial was omitted, and the left trials reported a wide 95% CI, leading to a wide 95% CI of the overall effect. In terms of the outcomes “freedom from TLR at the 6-month follow-up” and “primary patency”, there were more reasons. Although both overall effects were positive, most trials in the analysis were not completely supportive, with a 95% CI of the OR of 1. The FAIR and Liao trials were the only ones to report a positive effect of the outcome, and the conclusion changed when they were omitted (**Supplementary Fig. 13B,D**).

Upon summarizing the four outcomes, the bounds of the 95% CI of the OR for all four outcomes were very close to 1. Although we could explain the unstable effect of every outcome, the primary cause was limited data. In this context, a slight difference was observed. Particularly, in the outcome “primary patency”, which was reported by only three trials [13, 15, 17], once the Liao trial was omitted, the conclusion could turn to unsure from positive, although there was no heterogeneity across the three trials. Almost every trial mentioned the limitation of a small amount of participants, and we continued to urge the importance of a future multicenter randomized controlled trial with a large number of participants to ensure a powerful conclusion.

The majority of trials set endpoints 12 months after interventions. The pooled effects of the outcomes reflected an evident trend. The OR of the 6- and 12-month follow-ups was 0.22 versus 0.18 for the outcome “recurrent restenosis”, 2.70 versus 4.02 for the outcome “freedom from TLR”, 1.87 versus 2.38 for the outcome “clinical improvement”, and 0.71 versus 0.50 for the outcome “MAEs”. A similar trend is shown in Table 2, with a smaller change in the percentage of the PCBA group than that of the UCBA group. In the ISAR-PEBIS and COPA CABANA trials, researchers reported the outcome “freedom from TLR” at the 24-month follow-up, and the ratios of the PCBA and UCBA groups were 64.3% versus 44.8% and 48.1% versus 25.0%, respectively. The DEBATE-ISR trial reported a 36-month follow-up outcome of “freedom from TLR”, with a ratio of 59.1% in the PCBA group and 57.1% in the UCBA group [33]. Therefore, based on the above data, we concluded that PCBA might achieve better outcomes than UCBA and that the gap could be increased over time to up to 24 months. Long-time effectiveness is an important advantage of PCBA over other endovascular treatment methods, and more evidence is needed to extend the follow-up period.

## 5. Limitations

This evidence report has several limitations. First, there was little relevant data. There have been only seven prospective controlled trials of PCBA versus UCBA in the management of FP ISR since 2010. Among the chosen outcomes, only “freedom from TLR (12 months)” was reported by all seven trials, while the majority were referred to by fewer than five trials. The limited data was a key limitation of this meta-analysis.

Second, some trials reported a high missing follow-up rate. Almost half of the participants in the PACUBA trial were lost at the 12-month follow-up, which decreased the value of our analysis. In particular, in terms of the outcome “primary patency”, data from the PACUBA trial hardly impacted the overall effect. A high missing follow-up rate would sharply reduce the amount of data, leading to questionable conclusions.

Third, every study but the SFA ISR trial conducted dual-antiplatelet therapy for different durations, but only the DEBATE-ISR and FAIR trials reported some adverse events that might be related to the therapy. Relevant adverse events, such as ischemic and hemorrhagic accidents, should be completely reported.

Fourth, most trials focused on efficacy and safety outcomes; only the Liao and SFA ISR trials examined functional outcomes such as the Walking Impairment Questionnaire, EuroQol 5 dimensions quality-of-life measure, and 6-minute walking test. Intermittent claudication is always the primary symptom in patients with LEAD; thus, functional improvements should be assessed, especially in patients with walking impairments.

Finally, as discussed in the previous section, PCBA might achieve increasingly better outcomes than UCBA over time, but studies presenting such outcomes are scarce. However, the long-term durability and effects of PCBA are unknown. Although a cohort study assessed the clinical efficacy of DCB for FP lesions over 3 years [34], few studies have focused on the long-term durability and efficacy of PCBA versus UCBA for FP ISR.

## 6. Conclusions

Findings from our meta-analysis showed a reliable beneficial effect in terms of both angiographic and clinical success and a similar effect on the safety outcomes of PCBA versus UCBA. These data support clinician decisions regarding the management of FP ISR. Specifically, PCBA as a treatment strategy could achieve better short-term outcomes than UCBA for FP ISR management, including potent recurrent restenosis-lowering and symptom-improving capacity without increased MAEs.

## Supplementary Material

Supplementary material associated with this article can be found, in the online version, at https://doi.org/10.31083/j.rcm2309315.

## References

[1] Bartelink ML (2018). Epidemiology and risk factors. Oxford Medicine Online. https://oxfordmedicine.com/view/10.1093/med/9780198784906.001.0001/med-9780198784906-chapter-775.

[2] Maleckis K, Anttila E, Aylward P, Poulson W, Desyatova A, MacTaggart J (2018). Nitinol Stents in the femoropopliteal artery: a mechanical perspective on material, design, and performance. *Annals of Biomedical Engineering*.

[3] Aboyans V, Ricco JB, Bartelink MEL, Björck M, Brodmann M, Cohnert T (2018). 2017 ESC Guidelines on the diagnosis and treatment of peripheral arterial diseases, in collaboration with the European Society for Vascular Surgery (ESVS): Document covering atherosclerotic disease of extracranial carotid and vertebral, mesenteric, renal, upper and lower extremity arteriesEndorsed by: the European Stroke Organization (ESO)The Task Force for the Diagnosis and Treatment of Peripheral Arterial Diseases of the European Society of Cardiology (ESC) and of the European Society for Vascular Surgery (ESVS). *European Heart Journal*.

[4] Ho KJ, Owens CD (2017). Diagnosis, classification, and treatment of femoropopliteal artery in-stent restenosis. *Journal of Vascular Surgery*.

[5] Iida O, Takahara M, Soga Y, Hirano K, Yamauchi Y, Zen K (2016). The characteristics of in-stent restenosis after drug eluting stent implantation in femoropopliteal lesions and 1-year prognosis after repeat endovascular therapy for these lesions. *JACC: Cardiovascular Interventions*.

[6] Sobieszczyk P (2016). In-stent restenosis after femoropopliteal interventions with drug-eluting stents: same but different. *JACC: Cardiovascular Interventions*.

[7] Li J, Parikh SA (2017). Drug-coated balloons for long lesions in peripheral arterial disease. *The Journal of Cardiovascular Surgery*.

[8] Giacoppo D, Alfonso F, Xu B, Claessen BEPM, Adriaenssens T, Jensen C (2020). Paclitaxel-coated balloon angioplasty vs. drug-eluting stenting for the treatment of coronary in-stent restenosis: a comprehensive, collaborative, individual patient data meta-analysis of 10 randomized clinical trials (DAEDALUS study). *European Heart Journal*.

[9] Page MJ, McKenzie JE, Bossuyt PM, Boutron I, Hoffmann TC, Mulrow CD (2021). The PRISMA 2020 statement: an updated guideline for reporting systematic reviews. *International Journal of Surgery*.

[10] Cumpston M, Li T, Page MJ, Chandler J, Welch VA, Higgins JP (2019). Updated guidance for trusted systematic reviews: a new edition of the Cochrane Handbook for Systematic Reviews of Interventions. *The Cochrane Database of Systematic Reviews*.

[11] Liistro F, Angioli P, Porto I, Ricci L, Ducci K, Grotti S (2014). Paclitaxel-eluting balloon vs. standard angioplasty to reduce recurrent restenosis in diabetic patients with in-stent restenosis of the superficial femoral and proximal popliteal arteries: The DEBATE-ISR study. *Journal of Endovascular Therapy*.

[12] Krankenberg H, Tübler T, Ingwersen M, Schlüter M, Scheinert D, Blessing E (2015). Drug-coated balloon versus standard balloon for superficial femoral artery in-stent restenosis: the randomized femoral artery in-stent restenosis (FAIR) trial. *Circulation*.

[13] Kinstner CM, Lammer J, Willfort-Ehringer A, Matzek W, Gschwandtner M, Javor D (2016). Paclitaxel-eluting balloon versus standard balloon angioplasty in in-stent restenosis of the superficial femoral and proximal popliteal artery: 1-year results of the PACUBA trial. *JACC: Cardiovascular Interventions*.

[14] Ott I, Cassese S, Groha P, Steppich B, Voll F, Hadamitzky M (2017). ISAR‐PEBIS (Paclitaxel‐Eluting Balloon Versus Conventional Balloon Angioplasty for in‐Stent Restenosis of Superficial Femoral Artery): a Randomized Trial. *Journal of the American Heart Association*.

[15] Liao CJ, Song SH, Li T, Zhang Y, Zhang WD (2019). Randomized controlled trial of orchid drug-coated balloon versus standard percutaneous transluminal angioplasty for treatment of femoropopliteal artery in-scent restenosis. *International Angiology*.

[16] Tepe G, Schroeder H, Albrecht T, Reimer P, Diehm N, Baeriswyl J (2020). Paclitaxel-Coated Balloon vs Uncoated Balloon Angioplasty for Treatment of in-Stent Restenosis in the Superficial Femoral and Popliteal Arteries: the COPA CABANA Trial. *Journal of Endovascular Therapy*.

[17] Mena C (2014). Lutonix® drug coated balloon vs. standard balloon angioplasty for treatment of femoropopliteal in-stent restenosis (SFA ISR). Clinicaltrials.gov. https://clinicaltrials.gov/ct2/show/NCT02063672.

[18] Feldman DN, Armstrong EJ, Aronow HD, Gigliotti OS, Jaff MR, Klein AJ (2018). SCAI consensus guidelines for device selection in femoral-popliteal arterial interventions. *Catheterization and Cardiovascular Interventions*.

[19] Betala JV, Langan EM, LaBerge M (2014). Drug-Coated Percutaneous Balloon Catheters. *Critical Reviews in Biomedical Engineering*.

[20] Speck U, Stolzenburg N, Peters D, Scheller B (2016). How does a drug-coated balloon work? Overview of coating techniques and their impact. *The Journal of Cardiovascular Surgery*.

[21] Cremers B, Biedermann M, Mahnkopf D, Böhm M, Scheller B (2009). Comparison of two different paclitaxel-coated balloon catheters in the porcine coronary restenosis model. *Clinical Research in Cardiology*.

[22] Buszman PP, Tellez A, Afari ME, Peppas A, Conditt GB, Rousselle SD (2013). Tissue Uptake, Distribution, and Healing Response after Delivery of Paclitaxel via second-Generation Iopromide-Based Balloon Coating. *JACC: Cardiovascular Interventions*.

[23] Aboyans V, Criqui MH, Abraham P, Allison MA, Creager MA, Diehm C (2012). Measurement and Interpretation of the Ankle-Brachial Index. *Circulation*.

[24] Aboyans V, Ho E, Denenberg JO, Ho LA, Natarajan L, Criqui MH (2008). The association between elevated ankle systolic pressures and peripheral occlusive arterial disease in diabetic and nondiabetic subjects. *Journal of Vascular Surgery*.

[25] Potier L, Abi Khalil C, Mohammedi K, Roussel R (2011). Use and Utility of Ankle Brachial Index in Patients with Diabetes. *European Journal of Vascular and Endovascular Surgery*.

[26] Nativel M, Potier L, Alexandre L, Baillet-Blanco L, Ducasse E, Velho G (2018). Lower extremity arterial disease in patients with diabetes: a contemporary narrative review. *Cardiovascular Diabetology*.

[27] Herraiz-Adillo Á, Cavero-Redondo I, Álvarez-Bueno C, Pozuelo-Carrascosa DP, Solera-Martínez M (2020). The accuracy of toe brachial index and ankle brachial index in the diagnosis of lower limb peripheral arterial disease: a systematic review and meta-analysis. *Atherosclerosis*.

[28] Singh GD, Armstrong EJ, Laird JR (2014). Femoropopliteal in-stent restenosis: current treatment strategies. *The Journal of Cardiovascular Surgery*.

[29] Katsanos K, Spiliopoulos S, Kitrou P, Krokidis M, Karnabatidis D (2018). Risk of Death Following Application of Paclitaxel‐Coated Balloons and Stents in the Femoropopliteal Artery of the Leg: a Systematic Review and Meta‐Analysis of Randomized Controlled Trials. *Journal of the American Heart Association*.

[30] Schneider PA, Laird JR, Doros G, Gao Q, Ansel G, Brodmann M (2019). Mortality not Correlated with Paclitaxel Exposure: an Independent Patient-Level Meta-analysis of a Drug-Coated Balloon. *Journal of the American College of Cardiology*.

[31] Ouriel K, Adelman MA, Rosenfield K, Scheinert D, Brodmann M, Peña C (2019). Safety of Paclitaxel-Coated Balloon Angioplasty for Femoropopliteal Peripheral Artery Disease. *JACC: Cardiovascular Interventions*.

[32] Nordanstig J, James S, Andersson M, Andersson M, Danielsson P, Gillgren P (2020). Mortality with Paclitaxel-Coated Devices in Peripheral Artery Disease. *New England Journal of Medicine*.

[33] Grotti S, Liistro F, Angioli P, Ducci K, Falsini G, Porto I (2016). Paclitaxel-Eluting Balloon vs Standard Angioplasty to Reduce Restenosis in Diabetic Patients with in-Stent Restenosis of the Superficial Femoral and Proximal Popliteal Arteries: three-year results of the DEBATE-ISR study. *Journal of Endovascular Therapy*.

[34] Torsello G, Stavroulakis K, Brodmann M, Micari A, Tepe G, Veroux P (2020). IN.PACT Global Investigators, Three-year sustained clinical efficacy of drug-coated balloon angioplasty in a real-world femoropopliteal cohort. *Journal of Endovascular Therapy*.

