# Supplementary material for: Paclitaxel-Coated versus Uncoated Balloon for Femoropopliteal In-Stent Restenosis: A Systematic Review and Meta-Analysis
Source: Rev Cardiovasc Med. 2022 Sep 14;23(9):315. doi: 10.31083/j.rcm2309315 (PMC11262338; doi:10.31083/j.rcm2309315)
Supplement: Supplementary file 1 [file 2153-8174-23-9-315-s1.docx]

**Supplementary Materials**

Supplementary Table 1. Assessment of evidence within the recurrent restenosis outcome.

| **Recurrent Restenosis for femoropopliteal in-stent restenosis** | | | | | | |
| --- | --- | --- | --- | --- | --- | --- |
| **Patient or population:** patients with femoropopliteal in-stent restenosis **Settings:**  **Intervention:** Paclitaxel-coated balloon angioplasty (PCB group) or uncoated balloon angioplasty (UCB group) | | | | | | |
| **Outcomes** | **Illustrative comparative risks* (95% CI)** | | **Relative effect (95% CI)** | **No of Participants (studies)** | **Quality of the evidence (GRADE)** | **Comments** |
|  | Assumed risk | Corresponding risk |  |  |  |  |
|  | **Control** | **Recurrent Restenosis** |  |  |  |  |
| **recurrent restenosis (6 months)** Follow-up: 6 months | **Study population** | | **OR 0·23**  (0·13 to 0·38) | 281 (4 studies) | ⊕⊕⊕⊕ **high** |  |
|  | **561 per 1000** | **227 per 1000** (142 to 327) |  |  |  |  |
|  | **Moderate** | |  |  |  |  |
|  | **593 per 1000** | **251 per 1000** (159 to 356) |  |  |  |  |
| **recurrent restenosis (12 months)** Follow-up: 12 months | **Study population** | | **OR 0·18**  (0·1 to 0·34) | 224 (3 studies) | ⊕⊕⊕⊝ **moderate**^1^ |  |
|  | **709 per 1000** | **305 per 1000** (196 to 453) |  |  |  |  |
|  | **Moderate** | |  |  |  |  |
|  | **667 per 1000** | **265 per 1000** (167 to 405) |  |  |  |  |
| *The basis for the **assumed risk** (e.g. the median control group risk across studies) is provided in footnotes. The **corresponding risk** (and its 95% confidence interval) is based on the assumed risk in the comparison group and the **relative effect** of the intervention (and its 95% CI).  **CI:** Confidence interval; **OR:** Odds ratio; | | | | | | |
| GRADE Working Group grades of evidence **High quality:** Further research is very unlikely to change our confidence in the estimate of effect.  **Moderate quality:** Further research is likely to have an important impact on our confidence in the estimate of effect and may change the estimate. **Low quality:** Further research is very likely to have an important impact on our confidence in the estimate of effect and is likely to change the estimate. **Very low quality:** We are very uncertain about the estimate. | | | | | | |
| ^1^ The DEBATE-ISR trial has a bias in the process, which may cause an error in the results. | | | | | | |

Supplementary Table 2. Assessment of evidence within the primary patency outcome.

| **Primary Patency for femoropopliteal in-stent restenosis** | | | | | | |
| --- | --- | --- | --- | --- | --- | --- |
| **Patient or population:** patients with femoropopliteal in-stent restenosis **Settings:**  **Intervention:** Paclitaxel-coated balloon angioplasty (PCB group) or uncoated balloon angioplasty (UCB group) | | | | | | |
| **Outcomes** | **Illustrative comparative risks* (95% CI)** | | **Relative effect (95% CI)** | **No of Participants (studies)** | **Quality of the evidence (GRADE)** | **Comments** |
|  | Assumed risk | Corresponding risk |  |  |  |  |
|  | **Control** | **Primary Patency** |  |  |  |  |
| **primary patency (12 months)** Follow-up: 12 months | **Study population** | | **OR 3·5**  (1·65 to 7·4) | 156 (3 studies) | ⊕⊕⊕⊝ **moderate**^1^ |  |
|  | **459 per 1000** | **748 per 1000** (583 to 863) |  |  |  |  |
|  | **Moderate** | |  |  |  |  |
|  | **478 per 1000** | **762 per 1000** (602 to 871) |  |  |  |  |
| *The basis for the **assumed risk** (e.g. the median control group risk across studies) is provided in footnotes. The **corresponding risk** (and its 95% confidence interval) is based on the assumed risk in the comparison group and the **relative effect** of the intervention (and its 95% CI).  **CI:** Confidence interval; **OR:** Odds ratio; | | | | | | |
| GRADE Working Group grades of evidence **High quality:** Further research is very unlikely to change our confidence in the estimate of effect.  **Moderate quality:** Further research is likely to have an important impact on our confidence in the estimate of effect and may change the estimate. **Low quality:** Further research is very likely to have an important impact on our confidence in the estimate of effect and is likely to change the estimate. **Very low quality:** We are very uncertain about the estimate. | | | | | | |
| ^1^ The PACUBA study contained a few number of participants in the 12-month follow-up, which may cause a low precision ratio. | | | | | | |

Supplementary Table 3. Assessment of evidence within the freedom from TLR outcome.

| **Freedom From TLR for femoropopliteal in-stent restenosis** | | | | | | |
| --- | --- | --- | --- | --- | --- | --- |
| **Patient or population:** patients with femoropopliteal in-stent restenosis **Settings:**  **Intervention:** Paclitaxel-coated balloon angioplasty (PCB group) or uncoated balloon angioplasty (UCB group) | | | | | | |
| **Outcomes** | **Illustrative comparative risks* (95% CI)** | | **Relative effect (95% CI)** | **No of Participants (studies)** | **Quality of the evidence (GRADE)** | **Comments** |
|  | Assumed risk | Corresponding risk |  |  |  |  |
|  | **Control** | **Freedom From TLR** |  |  |  |  |
| **Freedom from TLR (6 months)** Follow-up: 6 months | **Study population** | | **OR 2.55**  (1.25 to 5.19) | 391 (5 studies) | ⊕⊕⊕⊝ **moderate**^1^ |  |
|  | **839 per 1000** | **930 per 1000** (867 to 964) |  |  |  |  |
|  | **Moderate** | |  |  |  |  |
|  | **839 per 1000** | **930 per 1000** (867 to 964) |  |  |  |  |
| **Freedom From TLR (12 months)** Follow-up: 12 months | **Study population** | | **OR 4.02**  (2.55 to 6.34) | 475 (7 studies) | ⊕⊕⊕⊝ **moderate**^2^ |  |
|  | **613 per 1000** | **834 per 1000** (724 to 944) |  |  |  |  |
|  | **Moderate** | |  |  |  |  |
|  | **625 per 1000** | **850 per 1000** (737 to 962) |  |  |  |  |
| *The basis for the **assumed risk** (e.g. the median control group risk across studies) is provided in footnotes. The **corresponding risk** (and its 95% confidence interval) is based on the assumed risk in the comparison group and the **relative effect** of the intervention (and its 95% CI).  **CI:** Confidence interval; **RR:** Risk ratio; **OR:** Odds ratio; | | | | | | |
| GRADE Working Group grades of evidence **High quality:** Further research is very unlikely to change our confidence in the estimate of effect.  **Moderate quality:** Further research is likely to have an important impact on our confidence in the estimate of effect and may change the estimate. **Low quality:** Further research is very likely to have an important impact on our confidence in the estimate of effect and is likely to change the estimate. **Very low quality:** We are very uncertain about the estimate. | | | | | | |
| ^1^ Result of FAIR trial is not consistent with others. ^2^ The DEBATE-ISR trial has a bias in the process, which may cause an error in the results. | | | | | | |

Supplementary Table 4. Assessment of evidence within the clinical improvement outcome.

| **Clinical Improvement for femoropopliteal in-stent restenosis** | | | | | | |
| --- | --- | --- | --- | --- | --- | --- |
| **Patient or population:** patients with femoropopliteal in-stent restenosis **Settings:**  **Intervention:** Paclitaxel-coated balloon angioplasty (PCB group) or uncoated balloon angioplasty (UCB group) | | | | | | |
| **Outcomes** | **Illustrative comparative risks* (95% CI)** | | **Relative effect (95% CI)** | **No of Participants (studies)** | **Quality of the evidence (GRADE)** | **Comments** |
|  | Assumed risk | Corresponding risk |  |  |  |  |
|  | **Control** | **Clinical Improvement** |  |  |  |  |
| **clinical improvement (6 months)** Follow-up: 6 months | **Study population** | | **OR 1·86**  (1·1 to 3·16) | 276 (4 studies) | ⊕⊕⊕⊕ **high** |  |
|  | **479 per 1000** | **631 per 1000** (503 to 744) |  |  |  |  |
|  | **Moderate** | |  |  |  |  |
|  | **567 per 1000** | **709 per 1000** (590 to 805) |  |  |  |  |
| **clinical improvement (12 months)** Follow-up: 12 months | **Study population** | | **OR 2·38**  (1·49 to 3·8) | 337 (5 studies) | ⊕⊕⊝⊝ **low**^1,2^ |  |
|  | **540 per 1000** | **736 per 1000** (636 to 817) |  |  |  |  |
|  | **Moderate** | |  |  |  |  |
|  | **523 per 1000** | **723 per 1000** (620 to 806) |  |  |  |  |
| *The basis for the **assumed risk** (e.g. the median control group risk across studies) is provided in footnotes. The **corresponding risk** (and its 95% confidence interval) is based on the assumed risk in the comparison group and the **relative effect** of the intervention (and its 95% CI).  **CI:** Confidence interval; **OR:** Odds ratio; | | | | | | |
| GRADE Working Group grades of evidence **High quality:** Further research is very unlikely to change our confidence in the estimate of effect.  **Moderate quality:** Further research is likely to have an important impact on our confidence in the estimate of effect and may change the estimate. **Low quality:** Further research is very likely to have an important impact on our confidence in the estimate of effect and is likely to change the estimate. **Very low quality:** We are very uncertain about the estimate. | | | | | | |
| ^1^ The DEBATE-ISR trial has a bias in the process, which may cause an error in the results.  ^2^ Results of FAIR trial and Liao trial are a little different from those of the other three trials. | | | | | | |

Supplementary Table 5. Assessment of evidence within the ABI outcome.

| **ABI for femoropopliteal in-stent restenosis** | | | | | | |
| --- | --- | --- | --- | --- | --- | --- |
| **Patient or population:** patients with femoropopliteal in-stent restenosis **Settings:**  **Intervention:** Paclitaxel-coated balloon angioplasty (PCB group) or uncoated balloon angioplasty (UCB group) | | | | | | |
| **Outcomes** | **Illustrative comparative risks* (95% CI)** | | **Relative effect (95% CI)** | **No of Participants (studies)** | **Quality of the evidence (GRADE)** | **Comments** |
|  | Assumed risk | Corresponding risk |  |  |  |  |
|  | **Control** | **ABI** |  |  |  |  |
| **ABI (12 months)** Follow-up: 12 months |  | The mean ABI (12 months) in the intervention groups was **0.02 higher** (0.11 lower to 0.14 higher) |  | 216 (3 studies) | ⊕⊝⊝⊝ **very low**^1,2^ |  |
| *The basis for the **assumed risk** (e.g. the median control group risk across studies) is provided in footnotes. The **corresponding risk** (and its 95% confidence interval) is based on the assumed risk in the comparison group and the **relative effect** of the intervention (and its 95% CI).  **CI:** Confidence interval; | | | | | | |
| GRADE Working Group grades of evidence **High quality:** Further research is very unlikely to change our confidence in the estimate of effect.  **Moderate quality:** Further research is likely to have an important impact on our confidence in the estimate of effect and may change the estimate. **Low quality:** Further research is very likely to have an important impact on our confidence in the estimate of effect and is likely to change the estimate. **Very low quality:** We are very uncertain about the estimate. | | | | | | |
| ^1^ The COPA CABANA trial failed to present the ABI as they designed in the Method section. ^2^ This outcome is only published by 3 studies of 7 included studies, and the study Liao 2019 fail to keep consistent with the other two. | | | | | | |

Supplementary Table 6. Assessment of evidence within the MAEs outcome.

| **Major Adverse Events for femoropopliteal in-stent restenosis** | | | | | | |
| --- | --- | --- | --- | --- | --- | --- |
| **Patient or population:** patients with femoropopliteal in-stent restenosis **Settings:**  **Intervention:** Paclitaxel-coated balloon angioplasty (PCB group) or uncoated balloon angioplasty (UCB group) | | | | | | |
| **Outcomes** | **Illustrative comparative risks* (95% CI)** | | **Relative effect (95% CI)** | **No of Participants (studies)** | **Quality of the evidence (GRADE)** | **Comments** |
|  | Assumed risk | Corresponding risk |  |  |  |  |
|  | **Control** | **Major Adverse Events** |  |  |  |  |
| **major adverse event (6 months)** Follow-up: 6 months | **Study population** | | **OR 0·68**  (0·22 to 2·13) | 251 (3 studies) | ⊕⊕⊕⊝ **moderate**^1^ |  |
|  | **56 per 1000** | **38 per 1000** (13 to 111) |  |  |  |  |
|  | **Moderate** | |  |  |  |  |
|  | **43 per 1000** | **30 per 1000** (10 to 87) |  |  |  |  |
| **major adverse event (12 months)** Follow-up: 12 months | **Study population** | | **OR 0·51**  (0·27 to 0·96) | 321 (4 studies) | ⊕⊕⊕⊝ **moderate**^2^ |  |
|  | **196 per 1000** | **110 per 1000** (62 to 189) |  |  |  |  |
|  | **Moderate** | |  |  |  |  |
|  | **209 per 1000** | **119 per 1000** (67 to 202) |  |  |  |  |
| *The basis for the **assumed risk** (e.g. the median control group risk across studies) is provided in footnotes. The **corresponding risk** (and its 95% confidence interval) is based on the assumed risk in the comparison group and the **relative effect** of the intervention (and its 95% CI).  **CI:** Confidence interval; **OR:** Odds ratio; | | | | | | |
| GRADE Working Group grades of evidence **High quality:** Further research is very unlikely to change our confidence in the estimate of effect.  **Moderate quality:** Further research is likely to have an important impact on our confidence in the estimate of effect and may change the estimate. **Low quality:** Further research is very likely to have an important impact on our confidence in the estimate of effect and is likely to change the estimate. **Very low quality:** We are very uncertain about the estimate. | | | | | | |
| ^1^ Result of ISAR-PEBIS trial is not consistent with others. ^2^ The DEBATE-ISR trial has a bias in the process, which may cause an error in the results. | | | | | | |


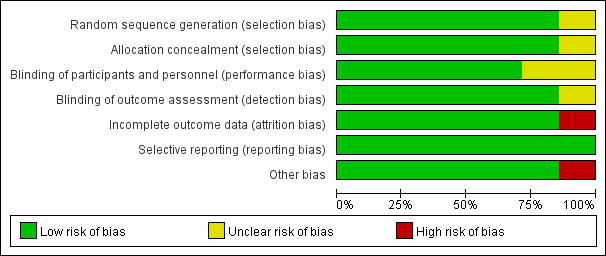


**Supplementary Fig. 1. Bias risk graph.** Judgements about each risk of bias item presented as percentages across all seven included studies.


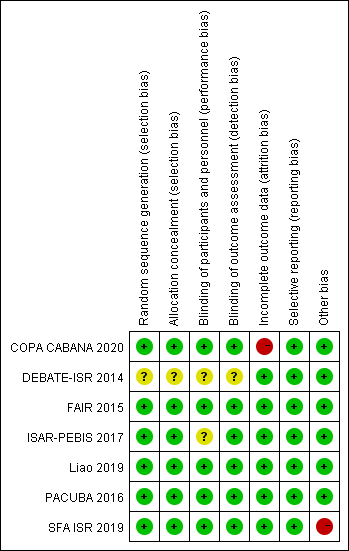


**Supplementary Fig. 2. Bias risk summary.** Judgements about each risk of bias item for each included study.


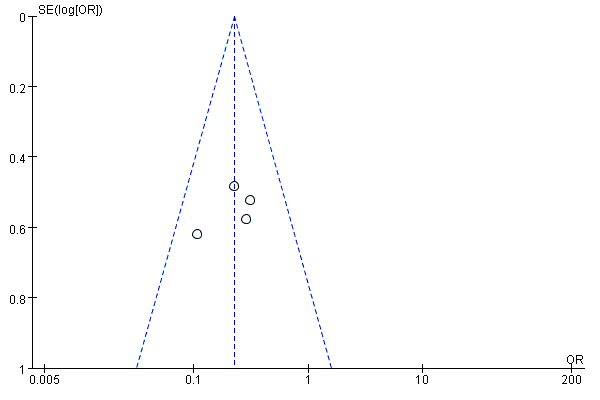


**Supplementary Fig. 3. Funnel plot estimated individual and overall effect of recurrent restenosis between PCB and UCB groups at 6 months.**


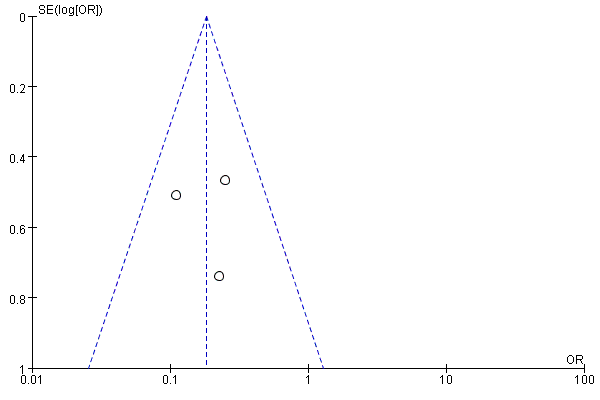


**Supplementary Fig. 4. Funnel plot estimated individual and overall effect of recurrent restenosis between PCB and UCB groups at 12 months.**


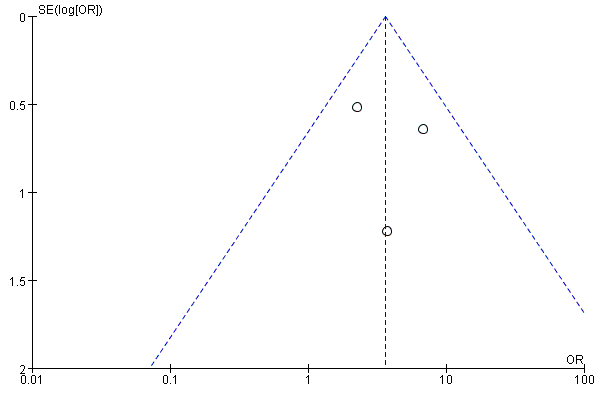


**Supplementary Fig. 5. Funnel plot of estimated individual and overall effect of primary patency between PCB and UCB groups at 12 months.**


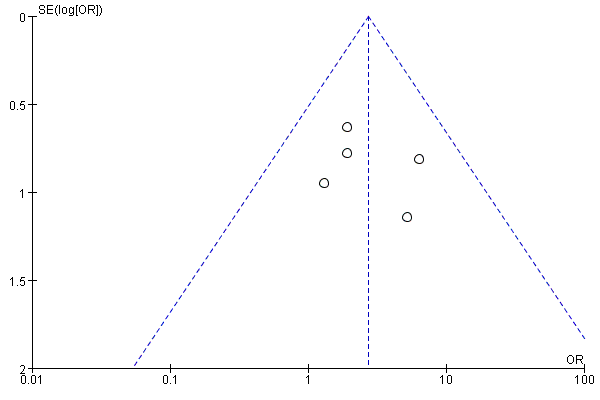


**Supplementary Fig. 6. Funnel plot of estimated individual and overall effect of freedom from TLR between PCB and UCB groups at 6 months.**

**
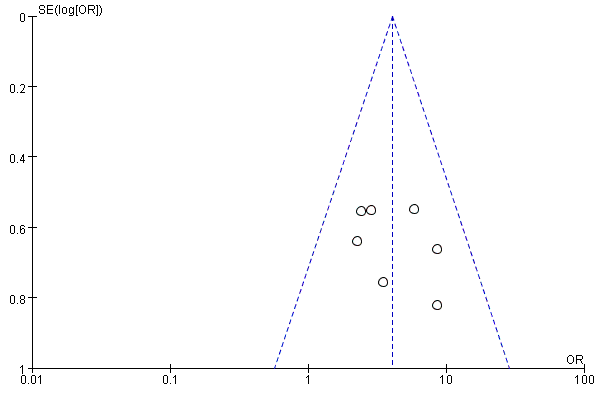
**

**Supplementary Fig. 7. Funnel plot of estimated individual and overall effect of freedom from TLR between PCB and UCB groups at 12 months.**

**
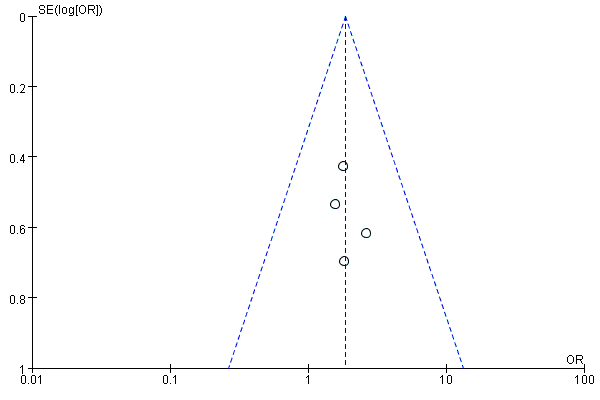
**

**Supplementary Fig. 8. Funnel plot of estimated individual and overall effect of clinical improvement between PCB and UCB groups at 6 months.**

**
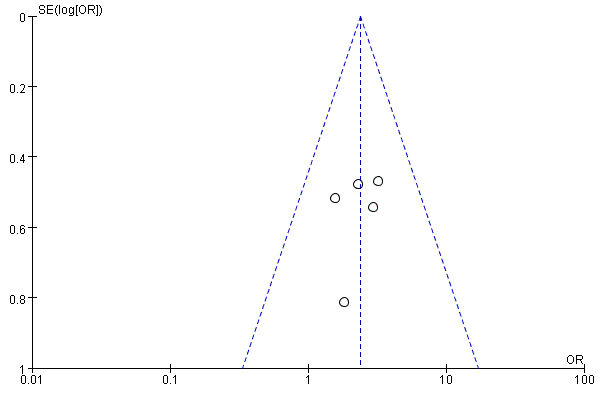
**

**Supplementary Fig. 9. Funnel plot of estimated individual and overall effect of clinical improvement between PCB and UCB groups at 12 months.**

**
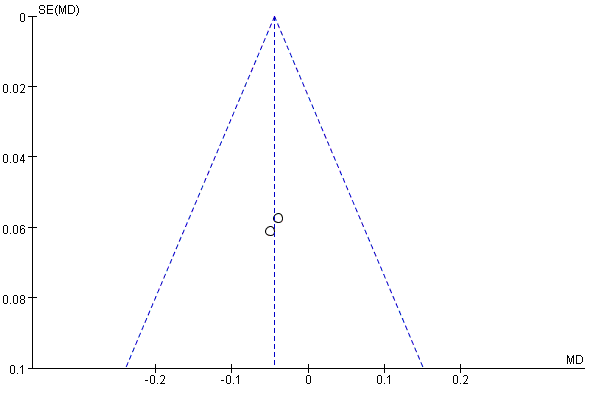
**

**Supplementary Fig. 10. Funnel plot of revised estimated individual and overall effect of the ABI between PCB and UCB groups at 12 months.**

**
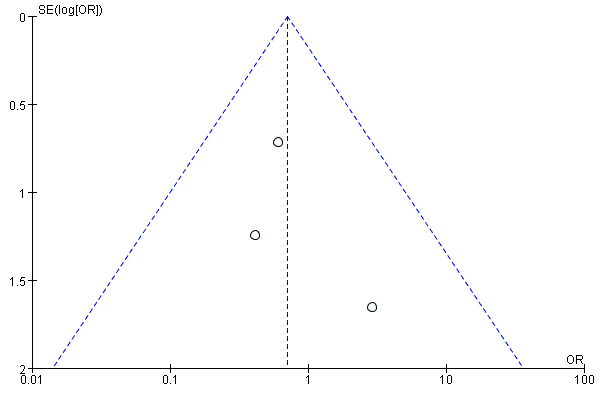
**

**Supplementary Fig. 11. Funnel plot of estimated individual and overall effect of MAEs between PCB and UCB groups at 6 months.**

**
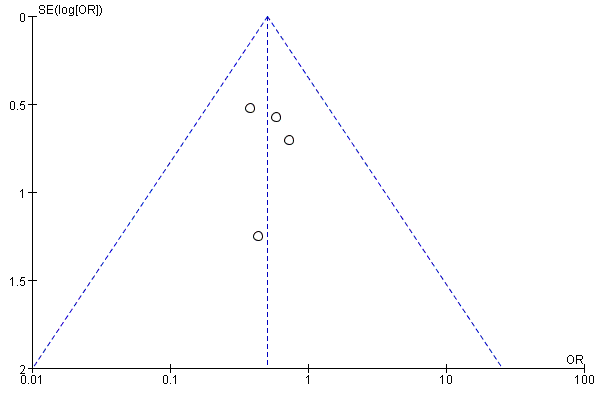
**

**Supplementary Fig. 12. Funnel plot of estimated individual and overall effect of MAEs between PCB and UCB groups at 12 months.**

**(A)**

**
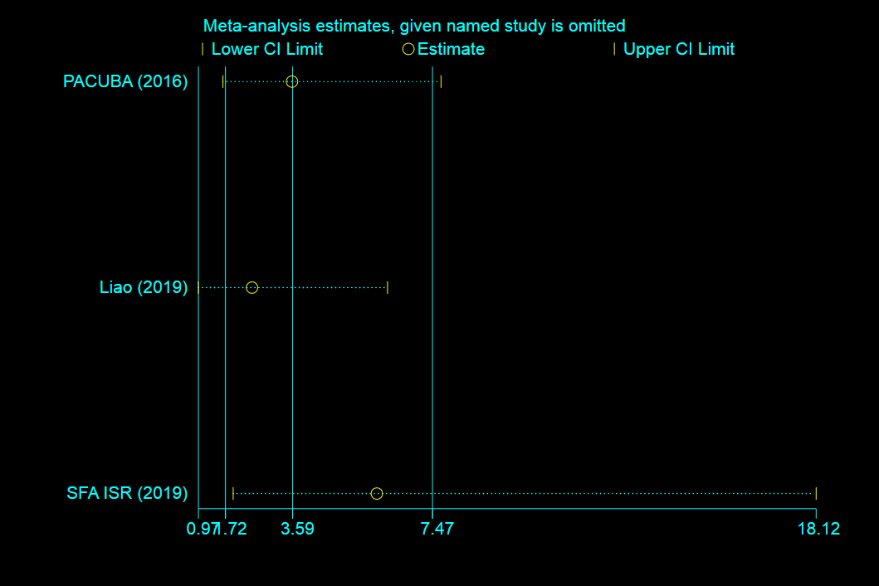
**

**(B)**

**
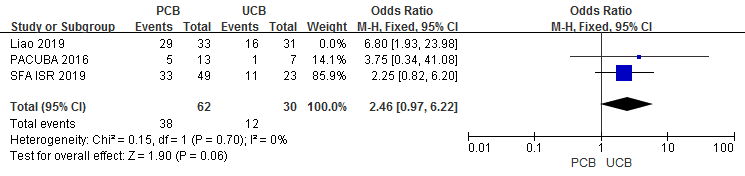
**

**(C)**

**
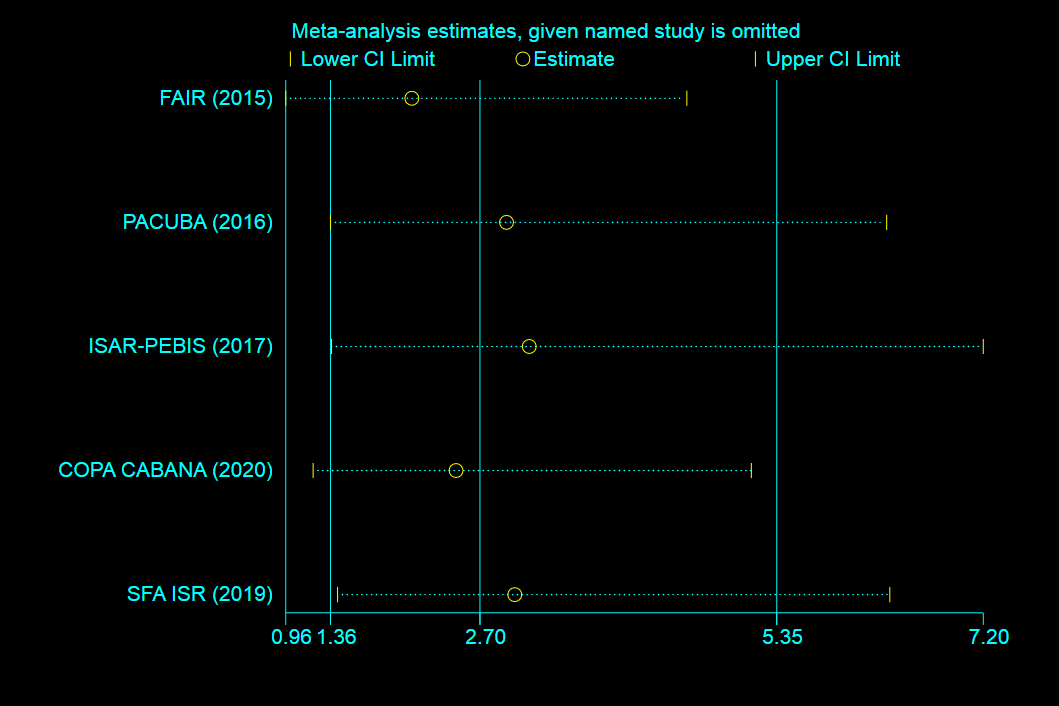
**

**(D)**

**
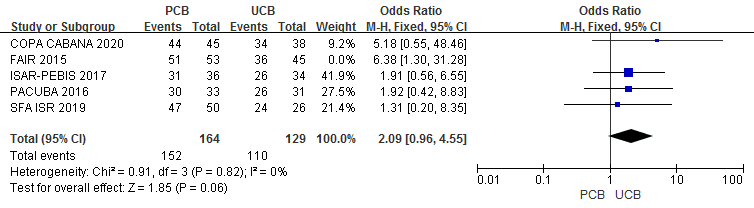
**

**(E)**

**
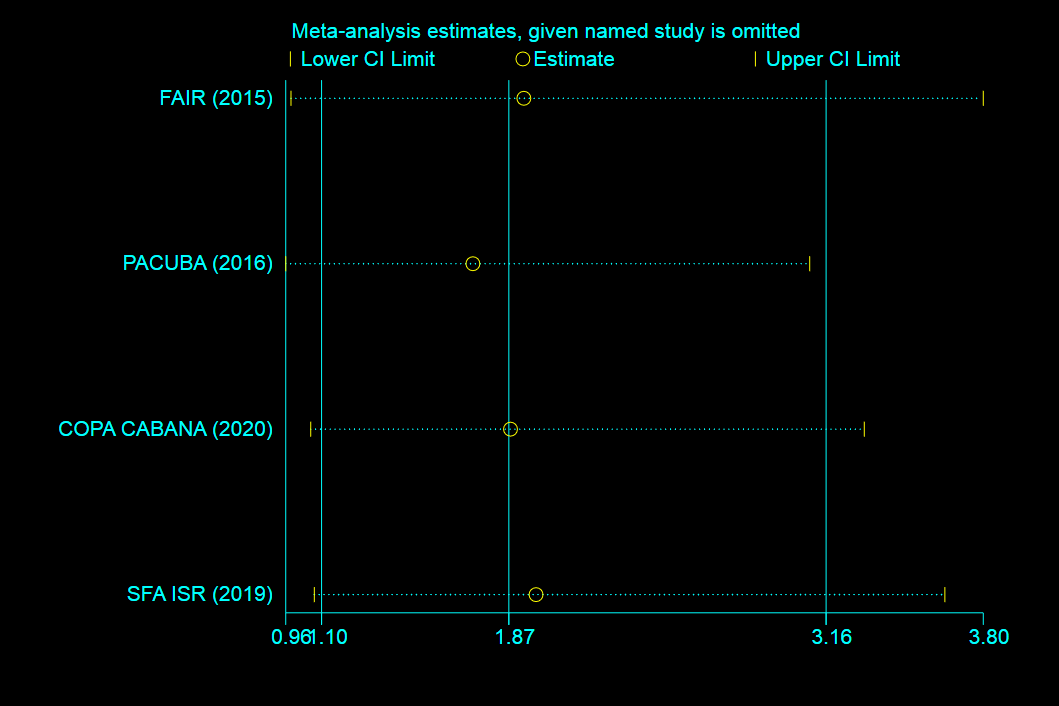
**

**(F)**

**
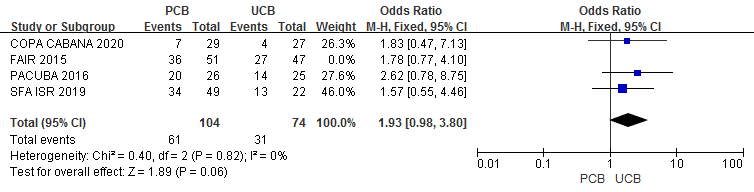
**

**(G)**

**
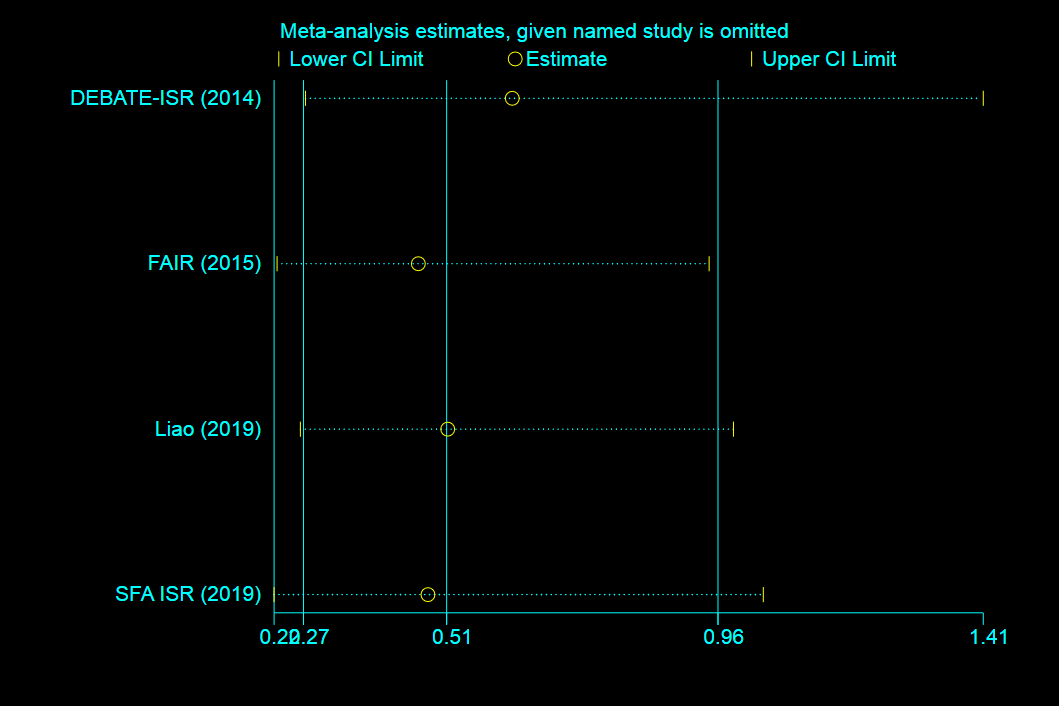
**

**(H)**

**
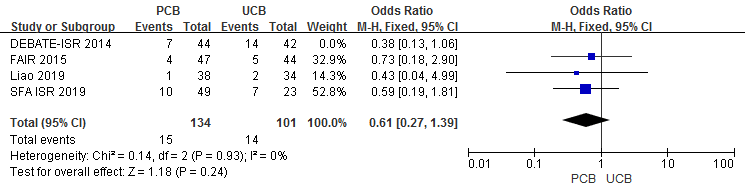
**

**Supplementary Fig. 13.** **Sensitivity analysis.** (A) Influence analysis of estimated individual and overall effect of the primary patency between PCB and UCB groups at 12 months. (B) Forest plot of estimated individual and overall effect of the primary patency between PCB and UCB groups at 12 months with Liao trial omitted. (C) Influence analysis of estimated individual and overall effect of the freedom from TLR at 6 months between PCB and UCB groups. (D) Forest plot of estimated individual and overall effect of the freedom from TLR at 6 months between PCB and UCB groups with FAIR trial omitted. (E) Influence analysis of estimated individual and overall effect of the clinical improvement between PCB and UCB groups at 6 months. (F) Forest plot of estimated individual and overall effect of the clinical improvement between PCB and UCB groups at 6 months with FAIR trial omitted. (G) Influence analysis of estimated individual and overall effect of the MAEs between PCB and UCB groups at 12 months. (H) Forest plot of estimated individual and overall effect of the MAEs between PCB and UCB groups at 12 months with DEBATE-ISR trial omitted.
